# Guanidinium-rich cell-penetrating peptidomimetic–doxorubicin conjugates show linker-dependent, reduced acute toxicity to cardiomyocytes and normal breast epithelium while retaining activity against a HER2-positive breast cancer model (SKBR3 spheroids)

**DOI:** 10.3389/fchem.2026.1945333

**Published:** 2026-09-10

**Authors:** Wiktoria Mallek, Hanna Bublewicz, Gabriela Mirecka, Kajetan Godziątkowski, Agnieszka Piwkowska, Adam Lesner, Magdalena Wysocka

**Affiliations:** 1 Faculty of Chemistry, University of Gdańsk, Gdańsk, Poland; 2 Laboratory of Molecular and Cellular Nephrology, Mossakowski Medical Research Institute, Polish Academy of Sciences, Gdańsk, Poland

**Keywords:** 3D spheroid, breast cancer, cardiotoxicity, cell-penetrating peptidomimetic, click chemistry, doxorubicin, guanidinium, peptide-drug conjugate

## Abstract

Doxorubicin (DOX) is a cornerstone anthracycline in breast cancer therapy, but its use is constrained by cumulative cardiotoxicity. We asked whether conjugation to a guanidinium-rich cell-penetrating peptidomimetic widens the window between anticancer activity and cardiomyocyte toxicity, or merely rescales potency. DOX was linked by azide–alkyne click chemistry to an eight-guanidinium peptidomimetic, H-O2Oc-[Dap(GO_2_)]_8_-O2Oc-NH_2_, through either a hexynoyl linker (CuAAC; DOX-ACA-HEX-G8) or a dibenzocyclooctyne linker (SPAAC; DOX-ACA-DBCO-G8), so that the pair differs in a single variable. Both conjugates and free DOX were tested in three-dimensional spheroids of HER2-positive SKBR3, triple-negative MDA-MB-231, non-tumorigenic mammary epithelium (HB2) and human AC16 cardiomyocytes (0.5–50 μM; 24, 48 and 72 h; CellTiter-Glo 3D), and in a three-read-out control panel (LDH, ATP, DNA). Free DOX was potent but non-selective, killing SKBR3, HB2 and AC16 with comparable 72 h IC_50_ values (<0.5, 0.78 and <0.5 µM) and leaving no meaningful window. DOX-ACA-HEX-G8 matched DOX against SKBR3 at 48–72 h (IC_50_ < 0.5 µM) while being 15-fold (HB2) and >18-fold (AC16) less toxic to the non-malignant lines. Because the SKBR3 IC_50_ is censored for both DOX and DOX-ACA-HEX-G8, their selectivity indices cannot be compared; the window is therefore established directly at matched concentration, without extrapolation: at 0.5 µM/72 h free DOX left SKBR3 at 6% and AC16 at 14% of control (an 8-percentage-point separation), whereas DOX-ACA-HEX-G8 left SKBR3 at 32% and AC16 at 122% (90 percentage points). The linker-matched DOX-ACA-DBCO-G8 was less selective, showing the effect tracks with linker chemistry. In the triple-negative model, DOX-ACA-HEX-G8 gave no window at all, and DOX-ACA-DBCO-G8 only a marginal one (selectivity index 1.6 for AC16 and 2.3 for HB2, both versus MDA-MB-231), although both conjugates were internalized. Both produced a pronounced low-dose hormetic signal in the non-malignant lines; a cell-free spike-recovery control excluded an artifact of the luminescence read-out, and the biomass read-out excluded proliferation, so the effect is metabolic and its mechanism remains open. DOX-ACA-HEX-G8 is a lead for anthracycline delivery with reduced acute cardiomyocyte toxicity, contingent on mechanistic studies of uptake and drug release.

## 1 Introduction

Breast cancer remains the most frequently diagnosed malignancy in women worldwide (Bray et al., 2024), and anthracyclines such as doxorubicin (DOX) are still among the most effective cytotoxic agents used across molecular subtypes (Minotti et al., 2004). DOX intercalates into DNA and poisons topoisomerase II, generating double-strand breaks, and it additionally drives the formation of reactive oxygen species. Two features limit its clinical value: cumulative, frequently irreversible cardiotoxicity mediated in large part by topoisomerase II-beta and oxidative injury in cardiomyocytes (Sawyer, 2013; Zhang S. et al., 2012), and the emergence of multidrug resistance driven by ATP-binding cassette (ABC) efflux transporters (Robey et al., 2018).

Strategies to widen the therapeutic index of DOX include liposomal formulations, antibody–drug conjugates, and peptide-based delivery. Among peptide carriers, arginine-rich and, more generally, guanidinium-rich cell-penetrating transporters are attractive because their polycationic guanidinium groups form bidentate hydrogen bonds with cell-surface anions and promote energy-dependent and -independent membrane translocation of otherwise poorly permeant cargo (Hao et al., 2022; Wender et al., 2008). Guanidinium-rich transporters have specifically been shown to help cargo evade efflux-mediated multidrug resistance (Vargas et al., 2014), providing a rationale for conjugating them to DOX.

**SCHEME 1:**
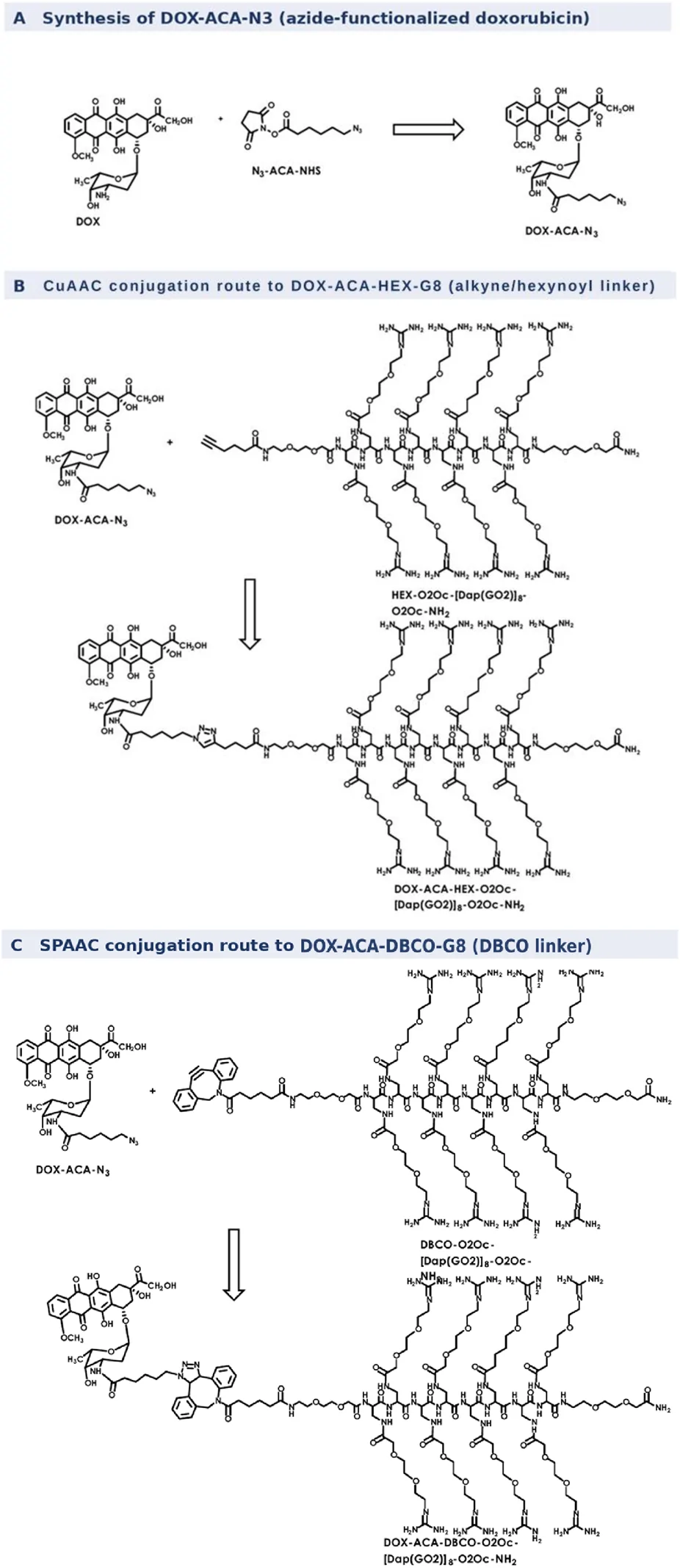
Panels A and B. **(A)** Synthesis of azide-functionalized doxorubicin (DOX-ACA-N_3_). **(B)** Copper-catalyzed azide–alkyne cycloaddition (CuAAC) of DOX-ACA-N_3_ to the alkyne/hexynoyl-terminated eight-guanidinium peptidomimetic to give DOX-ACA-HEX-G8. Panel C. **(C)** SPAAC click conjugation of DOX-ACA-N_3_ to the DBCO-terminated eight-guanidinium peptidomimetic to give DOX-ACA-DBCO-G8. The two conjugates differ in the linker and in the click chemistry (alkyne/CuAAC versus DBCO/SPAAC).

We previously synthesized peptidomimetics built from diaminopropionic acid (Dap) residues whose side chains carry guanidinium groups spaced by poly(ethylene glycol)-type O2Oc units (Romanowska et al., 2021; Romanowska et al., 2024), and conjugated them to DOX by azide–alkyne click chemistry (Scheme 1). The present study is a focused proof-of-concept built on the eight-guanidinium scaffold H-O2Oc-[Dap(GO_2_)]_8_-O2Oc-NH_2_, for which we have complete, high-quality 3D dose–response data. It deliberately compares only two conjugates that differ in a single variable, the linker chemistry (hexynoyl versus dibenzocyclooctyne), against a common free-DOX reference.

Two-dimensional monolayers poorly recapitulate the diffusion barriers, hypoxic gradients, dormant sub-populations, and anti-apoptotic microenvironment of solid tumors, and they systematically overstate drug sensitivity. Three-dimensional (3D) models are more predictive and reproducibly yield higher DOX IC_50_ values than matched 2D cultures, whether the geometry is a spheroid (Huang et al., 2020; Imamura et al., 2015) or a porous scaffold (Carbone et al., 2025). Crucially, conjugate studies frequently benchmark only against cancer cells and omit cardiomyocytes (e.g., (Ziaei et al., 2019)), so any claimed therapeutic advantage cannot be separated from generic potency. Here we pair two molecularly distinct breast cancer models, triple-negative MDA-MB-231 and HER2-positive SKBR3, with two non-malignant counterparts, non-tumorigenic mammary epithelium (HB2) and human cardiomyocytes (AC16), the latter enabling a direct cardiomyocyte read-out. Cardiomyocyte models are established for anthracycline injury, both in the AC16 line (Zhang et al., 2020) and in induced pluripotent stem cell–derived cardiomyocytes (Maillet et al., 2016). Our aim was to test whether guanidinium-rich conjugation genuinely widens the therapeutic window rather than simply rescaling potency.

## 2 Materials and methods

### 2.1 Compounds and selection rationale

Three test articles were studied: free doxorubicin hydrochloride (DOX·HCl; reference) and two conjugates of DOX with the eight-guanidinium peptidomimetic H-O2Oc-[Dap(GO_2_)]_8_-O2Oc-NH_2_, differing in the linker and the click chemistry used (Scheme 1): DOX-ACA-HEX-O2Oc-[Dap(GO_2_)]_8_-O2Oc-NH_2_ (alkyne/hexynoyl linker, prepared by copper-catalyzed azide–alkyne cycloaddition [CuAAC]; “DOX-ACA-HEX-G8”) and DOX-ACA-DBCO-O2Oc-[Dap(GO_2_)]_8_-O2Oc-NH_2_ (dibenzocyclooctyne linker, prepared by strain-promoted azide–alkyne cycloaddition [SPAAC]; “DOX-ACA-DBCO-G8”). These two conjugates were selected because they are the only test articles for which a complete, high-quality 3D dose–response dataset was available across all four cell lines and all three time points; restricting the analysis to a single-axis (linker) comparison at fixed guanidinium valency allows unambiguous interpretation.

Peptidomimetics were assembled by Fmoc solid-phase synthesis on the DAPEG platform reported previously (Romanowska et al., 2021); guanidinylation of Dap side chains, linker attachment (HEX-NHS or DBCO-NHS), attachment of the azide linker to DOX (DOX-ACA-N_3_), and the final click conjugation (CuAAC for DOX-ACA-HEX-G8; SPAAC for DOX-ACA-DBCO-G8) were monitored by UPLC. Conjugates were purified from post-reaction reagents by dialysis (Slide-A-Lyzer G3, 2 kDa MWCO, 0.25% acetic acid).

### 2.2 Analytical characterization

Conjugate identity was confirmed by MALDI-TOF-TOF mass spectrometry (Bruker Daltonics autoflex maX) and by reversed-phase UPLC, essentially as reported for DAPEG peptidomimetics (Romanowska et al., 2021). Chromatography used a Shimadzu Nexera X2 LC-30AD system with a Phenomenex Peptide XB-C18 column (150 × 2.1 mm, 1.7 µm); solvent A was 0.1% TFA in water and solvent B was 80% acetonitrile in A, with a linear gradient of 3%–90% B over 30 min at 0.3 mL/min. Detection was at 480 nm, the doxorubicin chromophore, and at 226 nm, the peptide-bond absorbance. Purity was determined as percent peak area of the 226 nm chromatogram; both conjugates were >95% pure. Physicochemical data are summarized in Table 1.

**TABLE 1:** Physicochemical characterization of the doxorubicin building block and the two conjugates (from MALDI-TOF-TOF mass spectrometry and UPLC; detection at 480 nm).

Compound	Click chemistry	Calc. molar mass (g/mol)	MS (MALDI-TOF-TOF)	UPLC tR (min)
DOX-ACA-N_3_ (building block)	— (amide coupling)	682.68	m/z 721.25 [M + K]^+^	11.05
DOX-ACA-HEX-O2Oc-[Dap(GO_2_)]_8_-O2Oc-NH_2_	CuAAC	3271.5	3270.5 [M] (K^+^ adduct series)	5.74
DOX-ACA-DBCO-O2Oc-[Dap(GO_2_)]_8_-O2Oc-NH_2_	SPAAC	3491.7	3491.4 [M]	18.41

Conjugates were purified by dialysis (Slide-A-Lyzer G3, 2 kDa MWCO, 0.25% acetic acid). UPLC: Shimadzu Nexera X2; Phenomenex Peptide XB-C18 (150 × 2.1 mm, 1.7 µm); 0.1% TFA/water (A) and 80% acetonitrile in A (B); linear gradient 3%–90% B over 30 min; 0.3 mL/min.

### 2.3 Cell lines and culture

MDA-MB-231 (triple-negative breast adenocarcinoma; ATCC) and SKBR3 (HER2-amplified breast adenocarcinoma) were maintained in high-glucose DMEM with 10% fetal bovine serum (FBS) and penicillin/streptomycin. HB2 (non-tumorigenic mammary luminal epithelial cells; Merck) were grown in high-glucose DMEM with 10% FBS, antibiotics, insulin (5 μg/mL) and hydrocortisone (5 μg/mL, added from an ethanolic stock), as described previously (Romanowska et al., 2024). AC16 human cardiomyocytes (Cytion) were maintained in DMEM:Ham’s F12 (1:1) supplemented with fetal bovine serum and penicillin/streptomycin. All cultures were kept at 37 °C in a humidified 5% CO_2_ atmosphere.

### 2.4 Three-dimensional spheroids and CellTiter-Glo 3D viability

Multicellular spheroids of MDA-MB-231, SKBR3, HB2, and AC16 were treated for 24, 48, and 72 h with compounds at 0.5, 1, 2, 5, 10, 20, and 50 µM. The full range was acquired for HB2 and AC16 at all three time points, for all four lines at 24 h, and for both conjugates on SKBR3 at 48 and 72 h. Two curves are bounded at 20 µM because the 50 µM point was not acquired at the later time points: MDA-MB-231 for all three compounds, and free DOX on SKBR3 (Supplementary Tables S1 and S2).

Compound stocks were prepared in aqueous ethanol (up to 50% v/v) and diluted so that a fixed small volume was added per well; the final per-well ethanol concentration was 1% (v/v) and was matched in the vehicle control. Reported non-cytotoxic ceilings for ethanol as a vehicle are cell-line- and exposure-dependent and span roughly 0.3%–1% (v/v) (Asiri et al., 2025; Ilieva et al., 2021; Kar et al., 2021; Landskroner and Tsai, 2025). The most finely resolved dose–response available places the transition at approximately 1% (v/v): across 0.005%–10% in fibroblasts, ethanol is non-cytotoxic up to 1% and cytotoxic above it, with mitochondrial cristae disruption and cytoskeletal disaggregation beginning at 1%, ATP falling to 81% ± 5% of control at 1% and 77% ± 16% at 3%, and apoptosis reaching ∼15% at 3% (Kar et al., 2021). Comparable ceilings are reported elsewhere. A maximum tolerated concentration of 1%–2% (v/v) has been described for keratinocyte, fibroblast and melanoma lines (Ilieva et al., 2021). A six-line cancer panel that included MDA-MB-231 lost more than 30% of viability at 0.3125% (v/v) within 24 h (Asiri et al., 2025). In bronchial epithelial cells the non-interference threshold falls from ≤0.5% (v/v) at 24 h to ≤0.25% at 48 h (Landskroner and Tsai, 2025).

The 1% (v/v) used here for exposures of up to 72 h therefore lies on the edge of the transition identified in the most detailed of these studies, and at every threshold reported for the longer exposures that carry the present conclusions. Consistent with this, the vehicle control itself reduced the AC16 DNA signal to 90% ± 7% of the untreated control (Section 3.7). All treated arms were vehicle-matched, so contrasts between arms remain internally controlled; the absolute viabilities of the non-malignant lines may nonetheless include a small solvent contribution and should be read accordingly.

Multicellular spheroids were formed by the nonadherent method as described previously (Mallek et al., 2025). Cells were seeded in 96-well U-bottom ultra-low-attachment plates (BIOFLOAT, Sarstedt) in the respective growth medium with 10% FBS and 1% penicillin–streptomycin, then cultured for 3 days at 37 °C in 5% CO_2_ to form spheroids before treatment (seeding densities: 750 cells per well for the breast-derived lines MDA-MB-231, SKBR3, and HB2, and 2000 cells per well for AC16 cardiomyocytes). Spheroid size was monitored by light microscopy at the assay time points. Mean diameter was 131–154 μm at 24 h, 297–321 μm at 48 h, and 333–367 μm at 72 h of exposure. From 48 h onward, the spheroids exceed the diameter at which oxygen and nutrient gradients are commonly reported to develop in tumor spheroids (Zanoni et al., 2016), so an oxygen-limited core cannot be excluded at 48 and 72 h and is not claimed to be absent.

Viability was quantified by CellTiter-Glo 3D (Promega) (Crouch et al., 1993; Zanoni et al., 2016), an ATP-dependent luminescence assay optimized for the mass-transfer properties of spheroids; luminescence was read on a CLARIOstar plate reader (BMG Labtech) and normalized to the untreated (0 µM) control set to 100%. CellTiter-Glo luminescence was verified against an ATP standard curve (simple linear regression, Y = 5.25 × 10^11^·[ATP] − 1.27 × 10^3^ RLU, with [ATP] in mol L^-1^; R^2^ = 0.99; Sy.x = 2.08 × 10^4^; slope 95% CI 5.01–5.49 × 10^11^; intercept not significantly different from zero), confirming assay linearity over the working range; the same ATP standard was applied to all four cell lines. Each concentration was tested in four to eight technical replicates per plate, in three independent biological experiments per cell line and time point. Normalized viabilities slightly below zero appear at the highest doses on the 72 h HB2 DOX curve (Supplementary Table S3); these arise when the treated luminescence falls to the level of the reagent blank and are reported as measured rather than truncated at zero. Each plate was normalized to its own control wells; controls from a different plate were not used, because plate controls differed substantially (for MDA-MB-231 at 72 h, 87 564 versus 60 510 RLU). At 24 h all three test articles were assayed on one plate; at 48 and 72 h the conjugates and free DOX were on separate plates.

Four exclusions were applied, all of them to the MDA-MB-231 dataset and to no other. On the 48 h conjugate plate, the whole of row C was removed because of a documented pipetting failure; the criterion was applied to the entire row and to both conjugates, irrespective of the values obtained. On the 48 h DOX plate, the column-12 control wells were excluded for a plate-edge effect, leaving F6–F7 as the control. That exclusion applies to that plate only and rests on the readings of that plate, not on a general rule: column-12 wells were retained as controls on the 24 h and 72 h plates, where no such effect was apparent. Two single wells were excluded as documented failures. The first, on the 72 h DOX plate, was row D at 10 μM, which read at untreated-control level while the four other wells of that group read 1%–7%, indicating that the compound had not been dispensed. The second, on the 72 h conjugate plate, was the DOX-ACA-HEX-G8 well in row B at 20 μM, which read 40% of control against 97%–136% for the other four. Each exclusion is applied at the level at which the failure occurred—the whole row where a row failed, the single well where one well failed—and never by reference to whether the resulting value supported the hypothesis. Individual data points, replicate values and the control wells used are given in the Supplementary Material (Supplementary Tables S1–S4).

### 2.5 IC_50_ estimation and selectivity index

**FIGURE 1:**
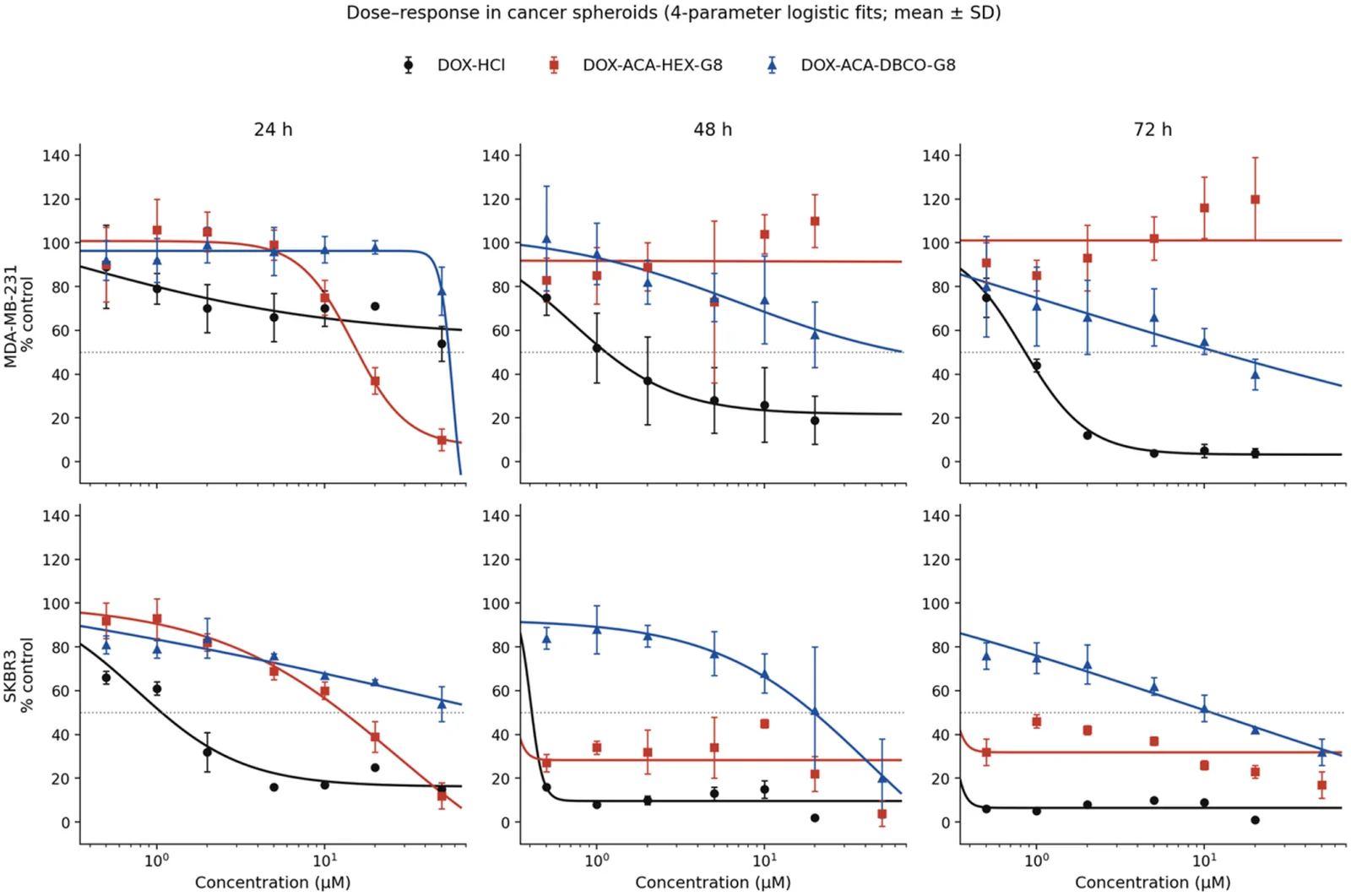
Dose–response of free DOX, DOX-ACA-HEX-G8, and DOX-ACA-DBCO-G8 in cancer spheroids (MDA-MB-231, top; SKBR3, bottom) at 24, 48, and 72 h. Points are mean ± SD of the replicate wells from three independent biological experiments (four to eight technical replicates per concentration in each); curves are four-parameter logistic fits. Dotted line, 50% viability. All panels were recomputed from the raw plate readings, each plate normalized to its own control wells (Section 2.4). MDA-MB-231 was tested to 20 μM at 48 and 72 h and to 50 μM at 24 h; SKBR3 was tested to 50 μM at all three time points (DOX to 20 μM at 48 and 72 h).

**FIGURE 2:**
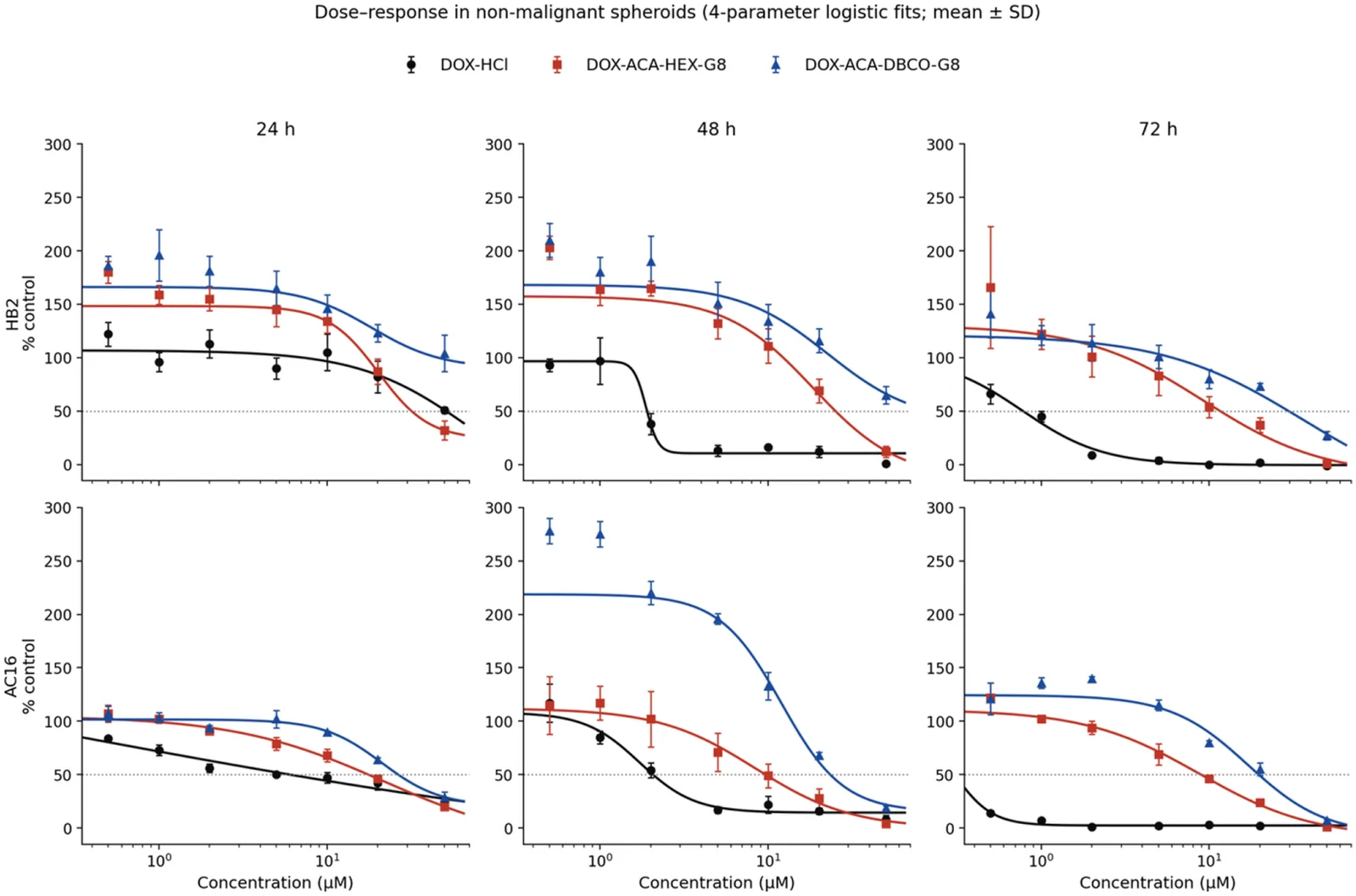
Dose–response in non-malignant spheroids (HB2, top; AC16 cardiomyocytes, bottom). Note the marked low-dose hormesis (apparent viability >100%) for both conjugates, most extreme for DOX-ACA-DBCO-G8 in AC16 at 48 h. Points are mean ± SD of the replicate wells from three independent biological experiments (four to eight technical replicates per concentration in each); curves are four-parameter logistic fits.

Normalized dose–response data (Supplementary Tables S1–S4) were fitted with a four-parameter logistic (variable-slope) model with free top and bottom asymptotes to accommodate low-dose hormesis. The absolute IC_50_ (concentration reducing viability to 50% of the untreated control) was determined from the fitted curve, and 95% confidence intervals were obtained by non-parametric bootstrap resampling of replicates (300 iterations). Values are reported as “>50” when the curve did not cross 50% within the tested range and as “<0.5” when the crossing lay below the lowest tested concentration (extrapolated) (Figures 1, 2; Table 2).

**TABLE 2:** IC_50_ values (µM; four-parameter logistic regression, 95% bootstrap CI in parentheses) from CellTiter-Glo 3D data. Three notations are used. “>50” and “>20” indicate that the fitted curve declined but did not reach 50% inhibition within the tested range; the upper limit of that range was 50 µM (24 h, all lines; 48 and 72 h, SKBR3, HB2 and AC16) or 20 µM (48 and 72 h, MDA-MB-231). “<0.5” indicates that the crossing lies below the lowest tested dose (0.5 µM) and is extrapolated. “n.i.” indicates no inhibition: apparent viability remained close to or above the untreated control at every concentration tested, so no IC_50_ exists (see Supplementary Table S1). MDA-MB-231 values were recomputed from the raw plate readings with each plate normalized to its own control wells (Section 2.4).

Cell line	Time	DOX	DOX-ACA-HEX-G8	DOX-ACA-DBCO-G8
MDA-MB-231 (TNBC)	24 h	>50	15.2 (14.1–16.7)	>50
​	48 h	1.06 (0.95–1.21)	n.i.	>20
​	72 h	0.94 (0.90–0.97)	n.i.	12.6 (8.3–15.5)
SKBR3 (HER2+)	24 h	1.07 (0.95–1.24)	13.0 (11.4–14.9)	>50
​	48 h	<0.5	<0.5	19.7 (11.2–44.9)
​	72 h	<0.5	<0.5	11.0 (8.59–14.0)
HB2 (normal breast)	24 h	>50	29.2 (23.9–36.9)	>50
​	48 h	1.84 (1.54–1.96)	24.9 (21.3–27.9)	>50
​	72 h	0.78 (0.67–0.88)	11.7 (8.68–13.8)	29.5 (26.7–31.6)
AC16 (cardiomyocyte)	24 h	6.00 (4.53–7.12)	17.6 (15.5–19.5)	26.5 (24.8–30.8)
​	48 h	2.07 (1.82–2.34)	9.64 (6.65–12.0)	23.4 (21.8–24.5)
​	72 h	<0.5	8.92 (7.84–9.85)	19.7 (18.0–21.9)

DOX-ACA-HEX-G8 = DOX-ACA-HEX-O2Oc-[Dap(GO_2_)]_8_-O2Oc-NH_2_; DOX-ACA-DBCO-G8 = DOX-ACA-DBCO-O2Oc-[Dap(GO_2_)]_8_-O2Oc-NH_2_. Values are from three independent biological experiments per cell line and time point, with four to eight technical replicates per concentration per plate. Bootstrap confidence intervals were obtained by resampling replicate wells and therefore describe within-experiment (technical) dispersion. An interval derived instead from the between-experiment spread of three IC_50_ values would itself rest on three points and be correspondingly unstable; the technical interval is therefore reported as a lower bound on the precision of each estimate and does not quantify between-experiment variability. Supplementary Tables S1–S4 report the mean and SD across the replicate wells of the three experiments at each concentration; per-experiment dose–response values are not tabulated separately.

Selectivity indices were computed as SI = IC_50_(non-malignant line)/IC_50_(cancer line). Where an IC_50_ was censored (below the lowest or above the highest tested concentration), the SI was not point-estimated; instead, the corresponding bound was propagated, using the lowest tested concentration (0.5 µM) or the highest tested concentration as the limiting value, and the SI is reported as a threshold. Where both IC_50_ values in a ratio were censored in the same direction, the SI is reported as not determinable.

Two indices that are both lower bounds cannot be compared, and censoring at a common limit does not imply equality of the censored values: at 0.5 µM/72 h free DOX reduced SKBR3 viability to 6% and DOX-ACA-HEX-G8 to 32% (Supplementary Table S2), so IC_50_(SKBR3, DOX) is materially lower than IC_50_(SKBR3, DOX-ACA-HEX-G8) even though both are reported as <0.5 µM. The fold-change in selectivity between the two compounds is therefore not determinable from these data and is not reported. Two quantities that are free of this limitation are reported instead: (i) the ratio of IC_50_ values on the same non-malignant line, which quantifies the reduction in toxicity relative to free DOX without any censored denominator (Table 3), and (ii) the difference in viability between a non-malignant line and SKBR3 at matched concentration, which uses measured values only (Table 4).

**TABLE 3:** Selectivity indices (SI = IC_50_ non-malignant/IC_50_ cancer) at 72 h. Higher is better; SI ≈ 1 indicates no window.

Comparison (72 h)	DOX	DOX-ACA-HEX-G8	DOX-ACA-DBCO-G8
AC16/SKBR3	n.d.^a^	>18	1.8
HB2/SKBR3	>1.6	>23	2.7
AC16/MDA-MB-231	<0.53	<0.45	1.6
HB2/MDA-MB-231	0.83	<0.58	2.3

Indices are computed from the 72 h IC_50_ values in Table 2. Where an IC_50_ is censored, the corresponding bound is propagated using the lowest tested concentration (0.5 µM) or, for a curve that does not cross 50% inhibition, the highest tested concentration, and the index is reported as a threshold rather than a point estimate.

^a^ Not determinable: both the AC16 and the SKBR3 IC_50_ for free DOX fall below the lowest tested concentration, so their ratio is unconstrained. Indices reported as thresholds cannot be compared with one another, and censoring at a common limit does not imply equality of the censored values: at 0.5 µM/72 h free DOX reduced SKBR3 viability to 6% and DOX-ACA-HEX-G8 to 32% (Supplementary Table S2), so the two SKBR3 IC_50_ values differ materially although both are reported as <0.5 µM. No fold-change in selectivity between compounds is therefore given. The two censoring-free comparisons are reported separately: the toxicity ratio on each non-malignant line relative to free DOX (AC16: >18-fold for DOX-ACA-HEX-G8 and >39-fold for DOX-ACA-DBCO-G8; HB2: 15-fold and 38-fold), and the matched-concentration separation in Table 4. Measured against cardiomyocytes alone, DOX-ACA-DBCO-G8 is the less toxic of the two conjugates; DOX-ACA-HEX-G8 reaches the larger index because it alone retains free-DOX-level activity against SKBR3. In the triple-negative comparison free DOX and DOX-ACA-HEX-G8 have no window, while DOX-ACA-DBCO-G8 has a modest one (1.6 and 2.3).

**TABLE 4:** Separation between non-malignant and HER2-positive spheroids at matched concentration, 72 h. Values are the difference in normalized viability, Δ = viability (non-malignant) − viability (SKBR3), in percentage points, computed from the measured points of Supplementary Tables S2–S4. A positive value means the non-malignant line is spared relative to SKBR3 at that concentration. This metric uses measured viabilities only and involves no curve fitting, no extrapolation and no censored IC_50_, so unlike the selectivity indices of Table 3 it is defined for every compound including free DOX.

Non-malignant line	Concentration	DOX	DOX-ACA-HEX-G8	DOX-ACA-DBCO-G8
AC16 (cardiomyocyte)	0.5 µM	+8	+90	+45
​	1 µM	+2	+56	+61
​	2 µM	−7	+52	+68
​	5 µM	−8	+32	+53
​	10 µM	−6	+20	+28
​	20 µM	+1	+1	+13
HB2 (normal breast)	0.5 µM	+60	+134	+65
​	1 µM	+40	+76	+46
​	2 µM	+1	+59	+42
​	5 µM	−6	+46	+39
​	10 µM	−9	+28	+28
​	20 µM	+1	+14	+31

Free DOX separates cardiomyocytes from SKBR3 by at most 8 percentage points and inverts the separation at 2–10 μM, i.e. it has no window at any tested concentration. DOX-ACA-HEX-G8 separates them by 90 points at 0.5 µM and maintains a separation to 10 µM. The 50 µM column is omitted because both lines are near the assay floor there and the difference is uninformative. Δ inherits the limitations of the underlying read-out: where the non-malignant viability exceeds 100% (Table 5), part of Δ reflects the signal elevation discussed in Sections 3.5, 3.9 and 4, whose mechanism is unresolved, so Δ should be read as an upper bound on the separation at 0.5–2 µM. The SKBR3 arm of Δ is unaffected by this, since no elevation was observed in SKBR3.

### 2.6 Fluorescence microscopy

**FIGURE 3:**
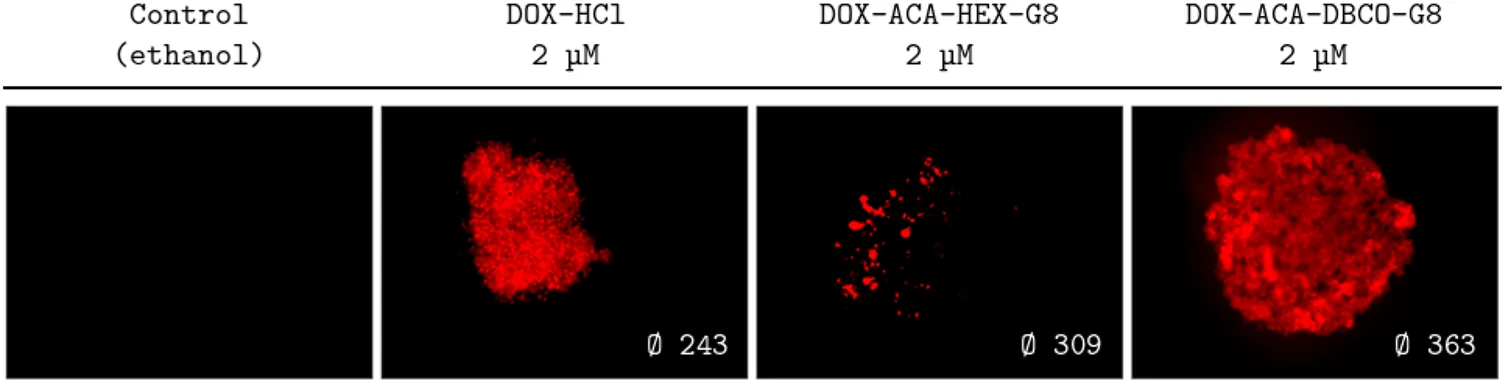
Cellular uptake by doxorubicin autofluorescence in SKBR3 at 24 h, compared at an equimolar, sub-lethal 2 µM. Panels: untreated control, free DOX, DOX-ACA-HEX-G8, and DOX-ACA-DBCO-G8. The red signal is the intrinsic fluorescence of doxorubicin and indicates intracellular drug accumulation; it is NOT a measure of viability or cell number. The untreated control is essentially signal-free, whereas all three DOX-bearing agents produce intracellular fluorescence, indicating that both conjugates are internalized. A single sub-lethal concentration is deliberately shown because fluorescence intensity scales with drug amount rather than cell number; a dose escalation would conflate drug loading with viability and must not be read as spheroid growth.

Treated SKBR3 and MDA-MB-231 cultures were imaged using fluorescence microscopy on an Olympus IX51, exploiting the intrinsic red fluorescence of doxorubicin, which reports cellular drug uptake and intracellular accumulation. Imaging was performed at 24, 48, and 72 h; because the purpose was to establish whether the conjugates enter cells at all, only the 24 h time point is analyzed here, at a concentration low enough to precede substantial cell loss. Representative SKBR3 fields at the 24 h time point (equimolar, sub-lethal 2 μM; with ethanol vehicle controls) are shown in Figure 3, and representative MDA-MB-231 fields in Supplementary Figure S1. Images were acquired and processed with CellSens Dimension software; identical acquisition and display settings (uniform autocontrast) were applied across panels for qualitative comparison.

### 2.7 Three-read-out control panel (LDH release, ATP, and DNA content)

To test whether the sparing of non-malignant cells requires covalent attachment of DOX, and to characterize the low-dose hormetic signal, a control panel with three orthogonal, microscopy-free read-outs was applied to AC16 cardiomyocytes and to HER2-positive SKBR3 cells at a single concentration (10 µM) for 72 h. The panel was run on three-dimensional spheroids formed, seeded and treated exactly as described in Section 2.4, so that its read-outs are directly comparable with the dose–response data. Spheroids were lysed *in situ* in the assay well without a wash step, so the DNA readout reflects the total double-stranded DNA retained in the well. Ten arms were compared: free DOX·HCl (A1); the linker–DOX intermediate DOX-ACA-N_3_ (A6); the two non-covalent DOX + carrier mixtures (A7: HEX-G8, A8: DBCO-G8); the two covalent conjugates DOX-ACA-HEX-G8 (A2) and DOX-ACA-DBCO-G8 (A3); the two bare carriers (A4: HEX-G8, A5: DBCO-G8); an ethanol vehicle control (K1); and an untreated control (K2). A maximal-lysis (death/max) control (K3) was included to define 100% LDH release.

Membrane lysis was quantified as lactate dehydrogenase (LDH) release with the CytoTox 96 Non-Radioactive Cytotoxicity Assay (Promega) according to the manufacturer’s instructions, and expressed as percent of the maximum (full-lysis) LDH signal (sample/maximum × 100), without subtraction of the spontaneous (untreated) signal; the untreated control therefore reports the low background release (∼3%) rather than 0%. Total double-stranded DNA content, used as a biomass read-out, was quantified with the Quant-iT PicoGreen dsDNA Assay Kit (Thermo Fisher) according to the manufacturer’s instructions. ATP and DNA signals were normalized, within each independent experiment, to that experiment’s own untreated control (K2) set to 100%; LDH was normalized within each experiment to that experiment’s maximal-lysis control (K3). Because the ethanol vehicle control (K1) itself reduces the DNA signal (Table 6), normalizing to K2 rather than K1 makes the reported values conservative across all treated arms. Each arm was tested in three independent experiments, each with three technical replicates; statistical analysis is described in Section 2.9. Per-experiment and per-replicate values are provided in the Supplementary Material (Supplementary Tables S5–S10).

### 2.8 Cell-free ATP spike-recovery control

The low-dose elevation of the CellTiter-Glo signal described in Section 3.5 could in principle arise from the detection chemistry rather than from the cells. Compound-dependent interference in luminescence assays is reproducible and concentration-dependent, which are the characteristics normally taken as evidence of genuine activity, and inhibition of firefly luciferase is among the most frequently encountered classes of such interference (Thorne et al., 2010). We therefore measured the recovery of a known quantity of ATP in the presence of each test article, in the absence of cells.

Because ATP and test article come into contact only when the reagent lyses the spheroids, the control was run as an immediate read: ATP, test article and reagent were combined and the plate was read without pre-incubation, reproducing the interval over which the two are in contact in the cell-based assay. Assays were run without cells in the medium and volume used for the AC16 spheroid experiments (DMEM:Ham’s F12 1:1 with 10% FBS), in a white 96-well flat-bottom plate, with the same CellTiter-Glo 3D reagent lot and the same CLARIOstar settings, so that the optical conditions of the luminescence read-out were reproduced.

Eight arms were compared: buffer alone; the ethanol vehicle at 1% (v/v); free DOX·HCl; the linker–DOX intermediate DOX-ACA-N_3_; the two bare carriers HEX-G8 and DBCO-G8; and the two covalent conjugates DOX-ACA-HEX-G8 and DOX-ACA-DBCO-G8. Each test article was assayed at 0.01, 0.05, 0.10, 0.25 and 0.50 µM, spanning the concentrations at which the elevation is observed; ethanol was held at 1% (v/v) in every well, as in the cell-based assays. A compound-only blank without ATP was included for every arm and concentration, so that any autoluminescence or background contributed by the test article was subtracted rather than assumed absent.

ATP was spiked at 50, 100, 230, 500 and 1,000 nM. These levels bracket the two quantities the control is intended to address: by the standard curve of Section 2.4, the untreated AC16 spheroid control corresponds to approximately 230 nM ATP, and the peak elevation observed in the non-malignant lines (278% of control; Section 3.5) to approximately 640 nM. Spanning the range also allows the form of any deficit to be identified, since a constant percentage across spike levels indicates a multiplicative effect whereas a deficit largest at the lowest spike indicates saturable binding of a fixed quantity of ATP.

Recovery was calculated as the background-corrected signal of the test arm divided by the background-corrected signal of the buffer arm at the same spike level, expressed as a percentage. ATP concentrations were back-calculated from the standard curve of Section 2.4. A recovery of 85%–115% was taken as the criterion for absence of interference, following common bioanalytical practice. Each combination of arm, concentration and spike level was measured once, giving 160 determinations across the design grid; the design therefore characterizes agreement across the grid rather than precision at any single point.

### 2.9 Statistics

Data are summarized as mean ± SD. Spheroid dose–response curves were fitted by four-parameter logistic regression with bootstrap 95% confidence intervals (Section 2.5). Each curve derives from three independent biological experiments; because the bootstrap resamples replicate wells, the resulting intervals describe technical rather than between-experiment dispersion. For the three-readout control panels, each arm was tested in three independent experiments (three technical replicates per experiment). Read-outs were first normalized within each experiment to that experiment’s own control (K2 for ATP and DNA; K3 for LDH), and all statistics were computed on the three resulting per-experiment means. Because the arms differ in dispersion by more than an order of magnitude (within-arm SD ranges from ∼0 to ∼23 percentage points), the homoscedasticity assumption of classical ANOVA is not met; arms were therefore compared by Welch one-way ANOVA followed by Games-Howell *post hoc* tests, which do not assume equal variances. No additional pairwise tests were applied, to avoid testing the same contrasts twice. Because n = 3 and several arms have a dispersion close to the reporting precision, p values are unstable in this regime and are reported only as thresholds; differences between arms are therefore described by the effect size (difference of normalized means) rather than by the p value alone. Raw per-experiment and per-replicate values are provided in the Supplementary Material (Supplementary Tables S5–S10) to enable direct assessment of dispersion.

## 3 Results

### 3.1 Synthesis and identity confirmation

Both eight-guanidinium conjugates were obtained by click conjugation of azide-functionalized doxorubicin (DOX-ACA-N_3_; calculated molar mass 682.68 g/mol; MALDI-TOF-TOF m/z 721.25 [M + K]+) to the corresponding peptidomimetic (Scheme 1). The alkyne/hexynoyl-terminated carrier was conjugated by CuAAC to give DOX-ACA-HEX-G8 (calc. 3271.5 g/mol; UPLC tR 5.74 min); MALDI-TOF-TOF gave m/z 3270.5 in the molecular-ion region, 1.0 Da below the calculated value, accompanied by a characteristic potassium-adduct series and an in-source fragment at m/z 2874.8 arising from cleavage of the doxorubicin O-glycosidic bond. The DBCO-terminated carrier was conjugated by SPAAC to give DOX-ACA-DBCO-G8 (calc. 3491.7 g/mol; MALDI-TOF-TOF observed [M] at m/z 3491.4; UPLC tR 18.41 min). Identity was confirmed by MALDI-TOF-TOF mass spectrometry and UPLC (Table 1), and the products were purified by dialysis. DOX-ACA-HEX-G8 and DOX-ACA-DBCO-G8, together with free DOX, were evaluated in the 3D spheroid panel (Figures 1, 2; Table 2).

### 3.2 Free doxorubicin is highly potent but non-selective in 3D spheroids

In 3D spheroids, free DOX reduced SKBR3 viability below 50% already at the lowest tested dose at 48–72 h (IC_50_ <0.5 µM). However, DOX was equally cytotoxic to the non-malignant lines, with 72 h IC_50_ values of 0.78 µM (HB2) and <0.5 µM (AC16 cardiomyocytes). Consequently, free DOX showed no meaningful therapeutic window. The AC16/SKBR3 index is not determinable, because both IC_50_ values fall below the lowest tested concentration, and the HB2/SKBR3 index is only a lower bound (>1.6) (Table 3). The absence of a window is nevertheless directly measurable at matched concentration: across 0.5–20 μM at 72 h, AC16 viability under free DOX never exceeded SKBR3 viability by more than 8 percentage points and was lower than it at 2–10 µM (Table 4). Against triple-negative MDA-MB-231 spheroids, DOX reached an IC_50_ of 1.06 µM (48 h) and 0.94 µM (72 h) but did not cross 50% inhibition at 24 h within the tested range (Figure 1).

### 3.3 DOX-ACA-HEX-G8 is selectively active against HER2-positive spheroids

Against SKBR3 spheroids, DOX-ACA-HEX-G8 matched free DOX at 48–72 h (IC_50_ <0.5 µM) after a slower onset at 24 h (13.0 µM, 95% CI 11.4–14.9). Against the non-malignant lines it was markedly less toxic (72 h IC_50_ 8.92 µM for AC16 and 11.7 µM for HB2). Because the SKBR3 IC_50_ for both DOX and DOX-ACA-HEX-G8 fell below the lowest tested concentration (<0.5 µM), the resulting 72 h selectivity indices can only be given as thresholds: >18 (AC16/SKBR3) and >23 (HB2/SKBR3) (Table 3). These thresholds cannot be compared with the corresponding indices for free DOX, which are themselves a lower bound (HB2/SKBR3 >1.6) or not determinable (AC16/SKBR3), and the fold-change in selectivity between the two compounds is therefore not reported (Section 2.5).

Two censoring-free statements carry the comparison instead. First, on each non-malignant line the conjugate is markedly less toxic than free DOX: 15-fold for HB2 (11.7/0.78) and >18-fold for AC16 (8.92/<0.5). Second, at matched concentration the separation between cardiomyocytes and tumor cells is present for the conjugate and absent for free DOX. At 0.5 µM and 72 h, DOX-ACA-HEX-G8 left SKBR3 at 32% and AC16 at 122% of control, a separation of 90 percentage points, whereas free DOX left SKBR3 at 6% and AC16 at 14%, a separation of 8 points. The separation persists to 10 µM for the conjugate and never appears for free DOX (Table 4). The absolute size of the window in IC_50_ terms can only be established with sub-micromolar dosing.

The DOX-ACA-DBCO-G8 comparator shares the guanidinium valency and differs only in the linker. It was less active against SKBR3 (72 h IC_50_ 11.0 µM, 95% CI 8.59–14.0) and correspondingly less selective (SI 1.8 and 2.7; both determinable, because neither IC_50_ is censored). This confirms that the selectivity is linker-dependent rather than a generic property of the guanidinium carrier (Figure 1, lower panels).

### 3.4 The conjugates do not benefit the triple-negative model

In contrast to SKBR3, MDA-MB-231 spheroids were substantially less sensitive to both conjugates than to free DOX. At 72 h, free DOX gave an IC_50_ of 0.94 µM, whereas the conjugates were far weaker. DOX-ACA-DBCO-G8 declined progressively and crossed 50% only at 72 h, giving an IC_50_ of 12.6 µM (95% CI 8.3–15.5), i.e. roughly 13-fold weaker than free DOX at the same time point; at 48 h it did not reach 50% within the tested range (IC_50_ > 20 µM). For DOX-ACA-HEX-G8 there was no inhibition at 48 or 72 h: apparent viability stayed between 73% and 110% at 48 h and between 85% and 120% at 72 h, never approaching 50%, so no IC_50_ exists at those time points and the response cannot be expressed as a fold-shift in potency relative to DOX (Table 2; Supplementary Table S1).

At 24 h the same compound gave a monotonic dose–response with an IC_50_ of 15.2 µM, reaching 10% of control at 50 µM (Supplementary Table S1). We cannot account for this. Regrowth of a culture that had been reduced to 37% of a proliferating control at 20 µM would have to overtake that control by 72 h, which is not plausible for a cytotoxic agent; the more parsimonious reading is that the high-dose signal elevation described in Section 3.5 is superimposed on the response at 48 and 72 h. The 24 and 48/72 h data were also acquired on different plates and the 50 µM dose that drove the 24 h response was not included at 48 and 72 h, so the two observations are not directly comparable. We report the discrepancy rather than resolve it.

Selectivity indices for MDA-MB-231 were below unity for DOX-ACA-HEX-G8 and for free DOX. DOX-ACA-DBCO-G8 reached 1.6 (AC16/MDA-MB-231) and 2.3 (HB2/MDA-MB-231), a window an order of magnitude smaller than the lower bounds DOX-ACA-HEX-G8 reaches against SKBR3 (Table 3). Fluorescence imaging showed that both conjugates are nonetheless internalized by MDA-MB-231 (Supplementary Figure S1), so the loss of activity in this subtype reflects events downstream of cellular uptake rather than a failure of delivery.

### 3.5 Both conjugates induce pronounced low-dose hormesis

At low concentrations, both conjugates increased the CellTiter-Glo signal above the untreated control in the non-malignant spheroids (Figure 2). The effect was largest in AC16 and HB2, but it was not confined to them. In MDA-MB-231 the same conjugate raised the signal above the untreated control at the high end of the range (104%–120% of control at 10–20 μM, 48 and 72 h; Supplementary Table S1) rather than at the low end. Stimulation at low and at high concentrations with a minimum in between is the profile we previously reported for the same DAPEG scaffold, using the same nonadherent spheroid protocol and the same CellTiter-Glo 3D read-out, in HEK-293T spheroids treated with N-acylated analogs: maxima at 1–2 μM and 20–50 µM and a minimum near 5–10 µM (Mallek et al., 2025). The signal elevation is therefore better regarded as a property of the carrier platform that is expressed in all four lines, differing in the concentration at which it appears, than as an effect restricted to the non-malignant models. Peak apparent viability at 48 h reached ≈ 278% of control (AC16, DOX-ACA-DBCO-G8, 0.5 µM) and ≈ 203–210% of control (HB2, both conjugates, 0.5 µM) (Table 5). This biphasic, hormesis-like response violates the assumptions of standard inhibition models and is the single largest source of uncertainty in the IC_50_ estimates; an assay-artifactual origin is excluded by the cell-free control reported in Section 3.9, and the biological basis of the effect remains unresolved (see Sections 4 and 5.2).

**TABLE 5:** Peak low-dose response in the non-malignant lines (maximum mean apparent viability, % of control, at ≤ 5 μM, 48 h). Free DOX is included so that the conjugates are compared against a reference run on the same lines and time point rather than against an implicit 100% baseline.

Line/compound	Peak viability	Concentration
AC16/DOX-ACA-DBCO-G8	278%	0.5 µM
AC16/DOX-ACA-HEX-G8	117%	1 µM
HB2/DOX-ACA-DBCO-G8	210%	0.5 µM
HB2/DOX-ACA-HEX-G8	203%	0.5 µM
AC16/DOX·HCl (free doxorubicin)	117%	0.5 µM
HB2/DOX·HCl (free doxorubicin)	97%	1 µM

Free DOX also exceeds the untreated control at the lowest doses (AC16 117%, HB2 97%), so a value modestly above 100% is not by itself specific to the conjugates; only the DOX-ACA-DBCO-G8 values (278% and 210%) and the DOX-ACA-HEX-G8 value on HB2 (203%) lie outside the range spanned by free DOX. The 117% reached by DOX-ACA-HEX-G8 on AC16 is identical to the free-DOX value on the same line and is not evidence of a conjugate-specific effect. Values are read from Supplementary Tables S3 and S4.

Apparent viability above 100% in an ATP-based assay must be confirmed by an orthogonal, non-ATP method before it is interpreted as proliferation or cytoprotection. The AC16/DOX-ACA-HEX-G8 value (117%) lies within the dispersion of the neighboring control points (Supplementary Table S4) and is listed for completeness only; it should not be read as a hormetic response. The remaining three values (203%–278%) exceed the control by a margin that the replicate dispersion does not account for.

### 3.6 Both conjugates are internalized (doxorubicin autofluorescence)

Because reduced 3D potency could in principle reflect failure of the bulky conjugates to enter cells, we imaged the intrinsic red fluorescence of doxorubicin in SKBR3 at an equimolar, sub-lethal 2 µM (24 h; Figure 3). The untreated control was signal-free. Free DOX and DOX-ACA-DBCO-G8 produced strong intracellular fluorescence. DOX-ACA-HEX-G8 produced a punctate signal that was clearly above the control but substantially weaker and more sparsely distributed than either comparator; on the fields shown, the fraction of the spheroid area above the red-channel threshold was 14% for free DOX, 30% for DOX-ACA-DBCO-G8 and 1.6% for DOX-ACA-HEX-G8, against 0.0% for the control. Because this signal reports intracellular drug accumulation and not viability, a single sub-lethal concentration was compared rather than a dose series (fluorescence intensity scales with drug loading, not cell number, and must not be interpreted as spheroid growth). The presence of intracellular fluorescence for both conjugates indicates that both are internalized, so a complete failure of uptake is excluded. The imaging does not, however, establish that they are internalized to a comparable extent. Quantitative uptake measurement (flow cytometry of dissociated spheroids with confocal colocalization) is required to further investigate this issue.

### 3.7 Control panel on non-malignant cells: covalent conjugation is required to spare cardiomyocytes

**FIGURE 4:**
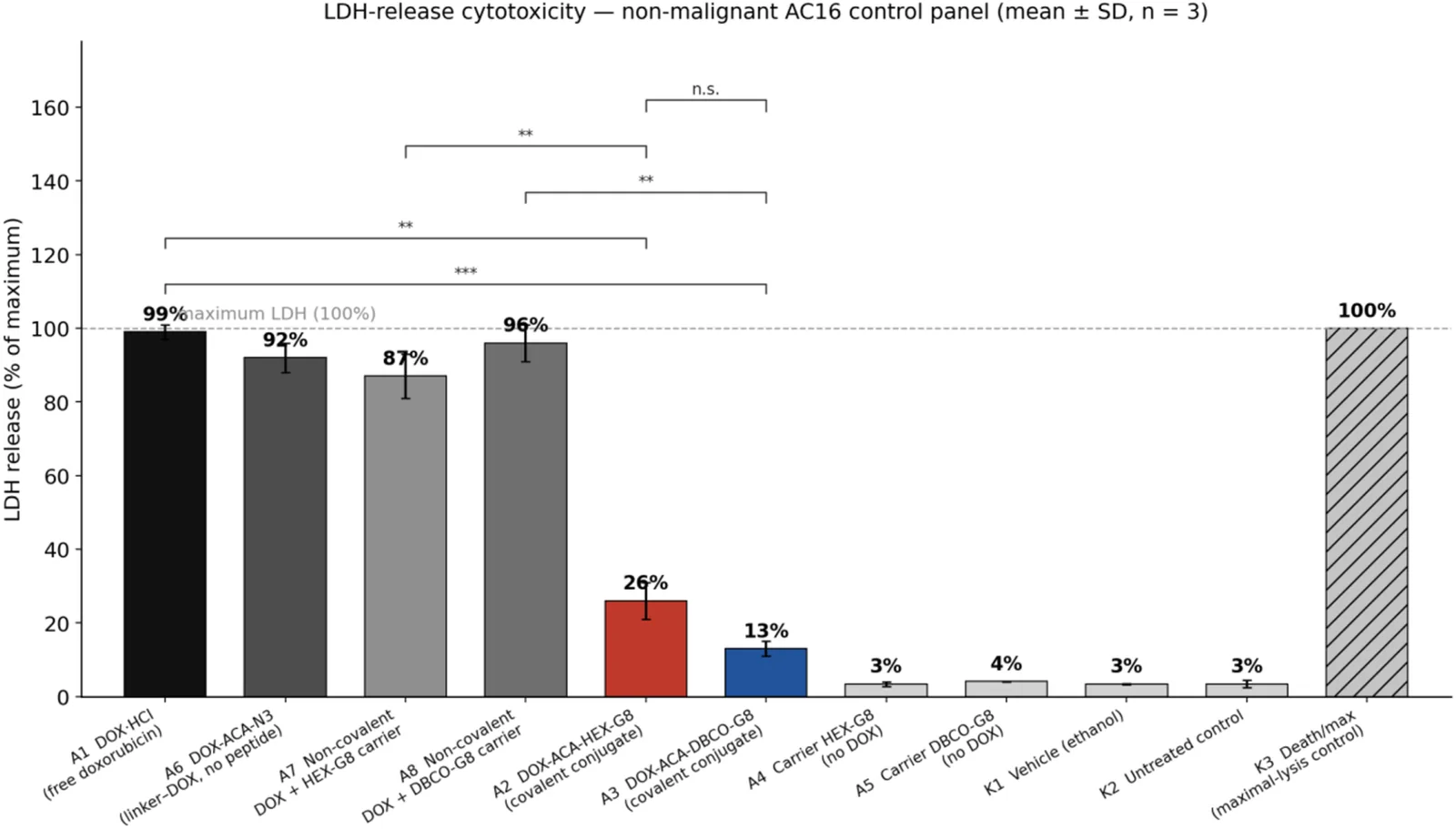
LDH release for the control panel on non-malignant cells (AC16 cardiomyocytes; single concentration 10 μM, 72 h; mean ± SD, n = 3 independent experiments), expressed as percent of the maximum (full-lysis) LDH signal (sample/maximum × 100, without subtraction of the spontaneous signal; the untreated control therefore reports the low background release, ∼3%). Free DOX, the DOX-ACA-N_3_ intermediate, and both non-covalent DOX + carrier mixtures give near-maximal LDH release (87%–99%), whereas the covalent conjugates give only 26% (DOX-ACA-HEX-G8) and 13% (DOX-ACA-DBCO-G8) and the bare carriers are inert (≤4%). On non-malignant cells, covalent conjugation, not mere co-administration, spares the cells from lytic damage. K3 (death/max) defines 100% release; K2 (untreated) and vehicle mark the assay floor (∼3%). Brackets show Games-Howell *post hoc* comparisons on the per-experiment means (***p < 0.001; **p < 0.01; *p < 0.05; n. s., p > 0.05); exact p values for every contrast are given in Supplementary Table S11. With n = 3 these p values are unstable and indicate only which contrasts the data support; the effect sizes carry the argument.

**FIGURE 5:**
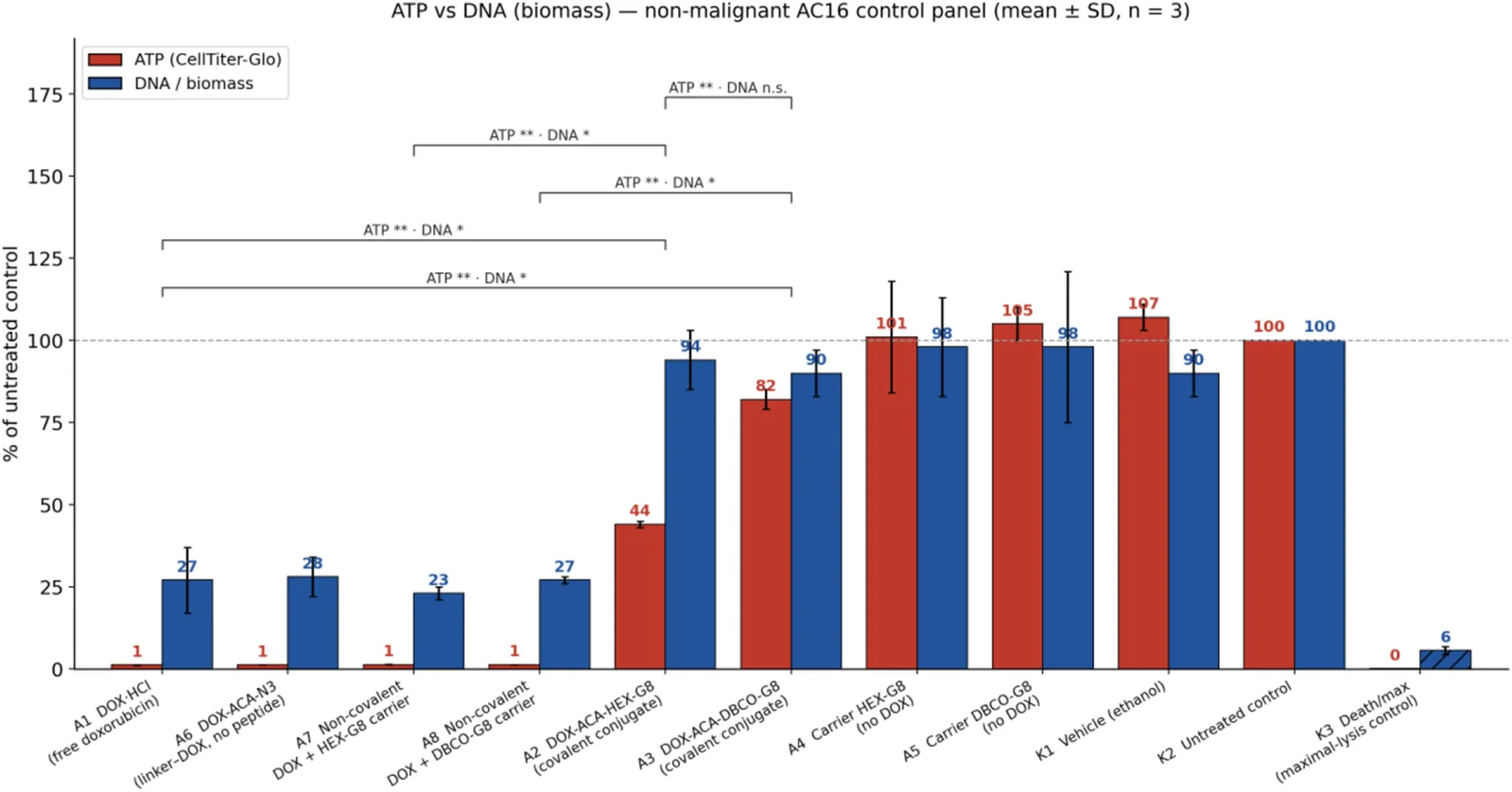
ATP versus DNA (biomass) for the control panel on non-malignant cells (AC16; 10 μM, 72 h; mean ± SD, n = 3), each normalized to untreated control. The cytotoxic arms (free DOX, DOX-ACA-N_3_, mixtures) fall to ∼1% (ATP) and ∼23–28% (DNA) and the carriers match the control. For the covalent conjugates, ATP drops below control (44% for DOX-ACA-HEX-G8, 82% for DOX-ACA-DBCO-G8) while DNA/biomass stays near control (∼90–94%); the ATP–biomass gap indicates reduced metabolic activity without proportional biomass loss at this single dose. K2 (untreated) is the 100% reference; K3 (death/max) marks the floor of both read-outs (ATP ∼0%, DNA ∼6%). Brackets show Games-Howell *post hoc* comparisons on the per-experiment means (***p < 0.001; **p < 0.01; *p < 0.05; n. s., p > 0.05); exact p values for every contrast are given in Supplementary Table S11. With n = 3 these p values are unstable and indicate only which contrasts the data support; the effect sizes carry the argument.

To test whether the reduced toxicity requires covalent attachment of DOX, and to determine the nature of the low-dose hormesis, we ran the full control panel on a non-malignant line (AC16 cardiomyocytes). Three orthogonal, microscopy-free read-outs were used: LDH release (membrane lysis), CellTiter-Glo (ATP), and DNA content (biomass), at a single concentration (10 μM, 72 h; three independent experiments; Figures 4, 5; Table 6; raw data in Supplementary Tables S5–S7). Free DOX, the linker–DOX intermediate DOX-ACA-N_3_, and both non-covalent DOX + carrier mixtures were strongly cytotoxic by all three read-outs (ATP ∼1% and DNA ∼23–28% of control; LDH ∼87–99%), whereas the covalent conjugates spared the cells (LDH 13%–26%; DNA 90%–94%) and the bare carriers were inert (≤4% LDH; ATP and DNA ∼100% of control). Three conclusions follow. First, covalent conjugation is required to spare non-malignant cells. A physical DOX + carrier mixture is as cytotoxic as free DOX, so co-administration does not protect; the effect is conferred by the peptidomimetic and not the linker (DOX-ACA-N_3_ is fully toxic). Against the biomass read-out, the conjugates leave AC16 cardiomyocytes essentially intact (DNA 90%–94% of control), in sharp contrast to the near-complete biomass loss caused by free DOX and the mixtures (DNA ∼23–28%).

**TABLE 6:** Control panel on non-malignant cells (AC16 cardiomyocytes; single concentration 10 μM, 72 h; mean ± SD of three independent experiments, three technical replicates each). Values are normalized within each experiment: LDH to that experiment’s maximal-lysis control K3 (sample/maximum × 100, without subtraction of the spontaneous signal), ATP and DNA to that experiment’s untreated control K2. Arms are listed in the order A1–A8, K1–K3. Entries marked “(ref.)” are the normalization reference for that read-out and are 100% by construction, with no dispersion; the same control carries a measured value in the read-outs it does not normalize. Every mean and SD in this table was recomputed from the per-replicate values of Supplementary Tables S5–S7 by the procedure of Sections 2.7 and 2.9: normalize within each experiment, then take the mean and SD of the three resulting per-experiment values.

Arm	Compound	LDH (% of max)	ATP (% ctrl)	DNA (% ctrl)
A1	DOX·HCl (free doxorubicin)	99 ± 2	1.2 ± 0.1	27 ± 10
A2	DOX-ACA-HEX-G8 (covalent conjugate)	26 ± 5	44 ± 1	94 ± 9
A3	DOX-ACA-DBCO-G8 (covalent conjugate)	13 ± 2	82 ± 3	90 ± 7
A4	Carrier HEX-G8 (no DOX)	3.4 ± 0.6	101 ± 17	98 ± 15
A5	Carrier DBCO-G8 (no DOX)	4.1 ± 0.1	105 ± 5	98 ± 23
A6	DOX-ACA-N_3_ (linker–DOX, no peptide)	92 ± 4	1.2 ± 0.0	28 ± 6
A7	Non-covalent DOX + HEX-G8 carrier	87 ± 6	1.3 ± 0.1	23 ± 2
A8	Non-covalent DOX + DBCO-G8 carrier	96 ± 5	1.3 ± 0.0	27 ± 1
K1	Vehicle (ethanol)	3.4 ± 0.2	107 ± 4	90 ± 7
K2	Untreated control	3.4 ± 1.0	100 (ref.)	100 (ref.)
K3	Maximal-lysis control (death/max)	100 (ref.)	0.1 ± 0.0	5.6 ± 1.2

Arms are grouped as cytotoxic references (A1, A6–A8), covalent conjugates (A2, A3), bare carriers (A4, A5) and controls (K1, K2). All three read-outs agree for the cytotoxic arms, which fall to ∼1% (ATP) and ∼23–28% (DNA) of the untreated control with near-maximal LDH release, whereas the covalent conjugates preserve biomass (DNA, 90%–94%) with low membrane lysis (13%–26%). For the covalent conjugates ATP is reduced (44% for DOX-ACA-HEX-G8 and 82% for DOX-ACA-DBCO-G8) without a proportional loss of biomass, indicating reduced metabolic activity rather than cell death at this dose. The ethanol vehicle control (K1) itself lowers DNA to 90% ± 7% of the untreated control, so a small solvent contribution to the AC16 read-outs cannot be excluded; all treated arms were vehicle-matched (Section 2.4).

Second, the two conjugates are not equivalent in cardiomyocytes, and their ranking is the opposite of that implied by the selectivity indices. DOX-ACA-HEX-G8 released twice as much LDH as DOX-ACA-DBCO-G8 (26% versus 13% of maximum) and reduced ATP roughly twice as far (44% versus 82% of control), while the two were indistinguishable on biomass (94% versus 90%; Games-Howell p = 1.0). The same ordering holds in the dose–response, where the 72 h AC16 IC_50_ is 8.92 µM for DOX-ACA-HEX-G8 and 19.7 µM for DOX-ACA-DBCO-G8, so that measured against cardiomyocytes alone DOX-ACA-DBCO-G8 is the less toxic compound (>39-fold less toxic than free DOX, versus >18-fold for DOX-ACA-HEX-G8). DOX-ACA-HEX-G8 is designated the lead not because it is kinder to cardiomyocytes than its comparator but because it is the only compound that retains free-DOX-level activity against SKBR3, and the separation it achieves rests on that potency.

Third, the 10 µM panel does not reproduce the low-dose ATP elevation and therefore cannot, on its own, characterize the hormesis. At 10 µM/72 h both conjugates reduced ATP below control (DOX-ACA-HEX-G8 ∼44%, DOX-ACA-DBCO-G8 ∼82%) while biomass was largely preserved (DNA ∼94% and ∼90%, respectively; ATP/DNA ≈ 0.5 and ≈ 0.9). These values agree with the spheroid dose–response measured on the same cell line at the same concentration and time point (AC16, 10 μM, 72 h: 46% and 80% of control). That is an independent check rather than a restatement, because CellTiter-Glo is itself an ATP read-out but was run on separate plates; and the 72 h AC16 IC_50_ values of 8.92 µM (DOX-ACA-HEX-G8) and 19.7 µM (DOX-ACA-DBCO-G8) independently place 10 μM at the midpoint of the first curve and well below the midpoint of the second. Thus, at this single high dose, ATP fell at least as fast as biomass rather than exceeding it, the opposite of the low-dose hormetic signal (up to ∼280% at 0.5 µM), which was not tested here; an artifact of the read-out is excluded in Section 3.9, and the mechanism is discussed in Section 4.

### 3.8 On HER2-positive cells the conjugates suppress net biomass accumulation with reduced membrane lysis

**FIGURE 6:**
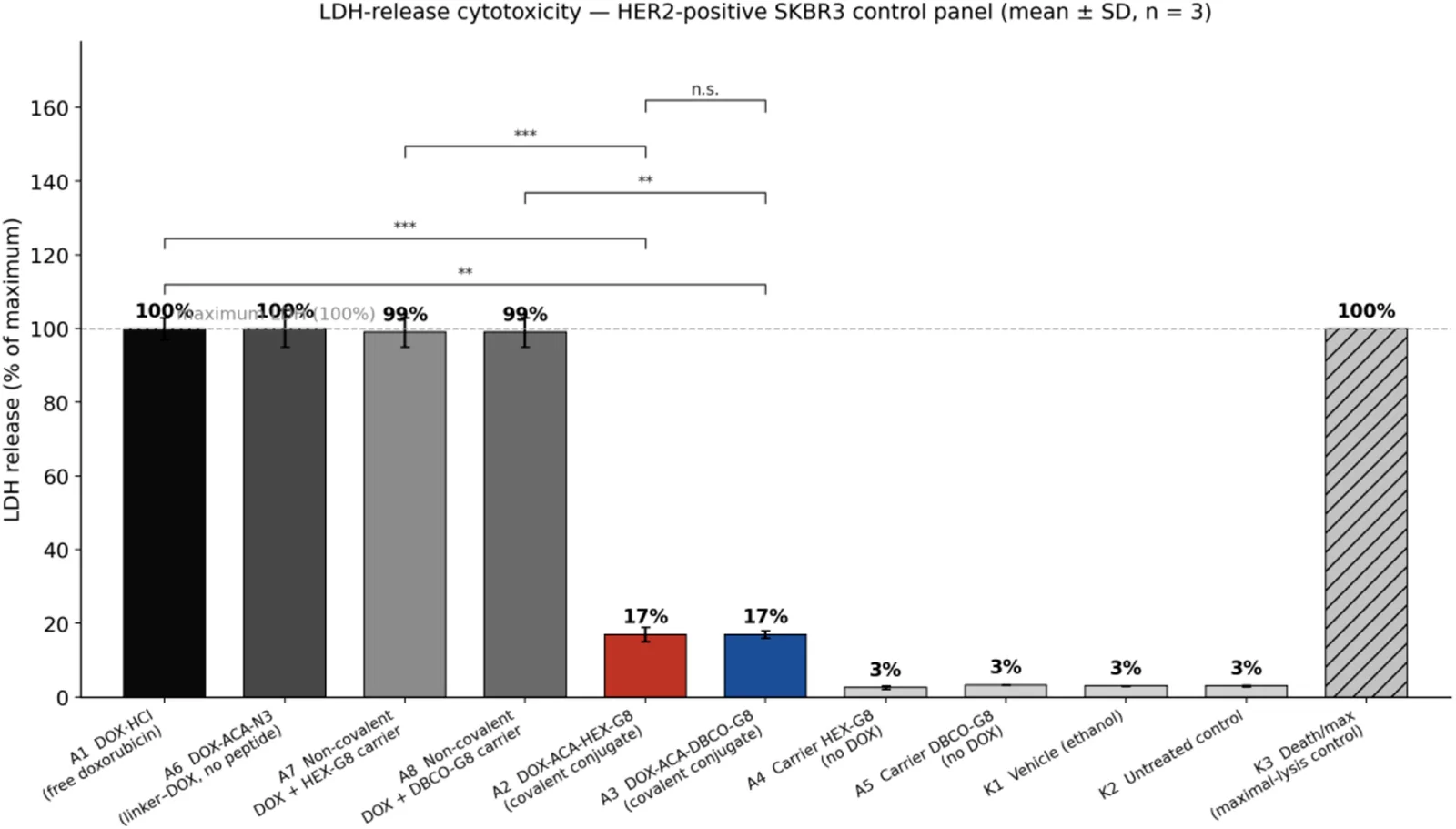
LDH release (% of maximum) on HER2-positive SKBR3 cells (10 μM, 72 h; mean ± SD, n = 3 independent experiments), expressed as sample/maximum × 100 without subtraction of the spontaneous signal. Free DOX, DOX-ACA-N_3_, and the non-covalent mixtures cause near-maximal release (∼100%), whereas the covalent conjugates release little LDH (∼17%) despite reducing biomass to ∼22% of control, indicating a non-lytic mode of action (apoptosis versus cytostasis pending caspase/annexin confirmation). K3 (death/max) defines 100% release; K2 (untreated) and vehicle mark the assay floor (∼3%). Brackets show Games-Howell *post hoc* comparisons on the per-experiment means (***p < 0.001; **p < 0.01; *p < 0.05; n. s., p > 0.05); exact p values for every contrast are given in Supplementary Table S11. With n = 3 these p values are unstable and indicate only which contrasts the data support; the effect sizes carry the argument.

**FIGURE 7:**
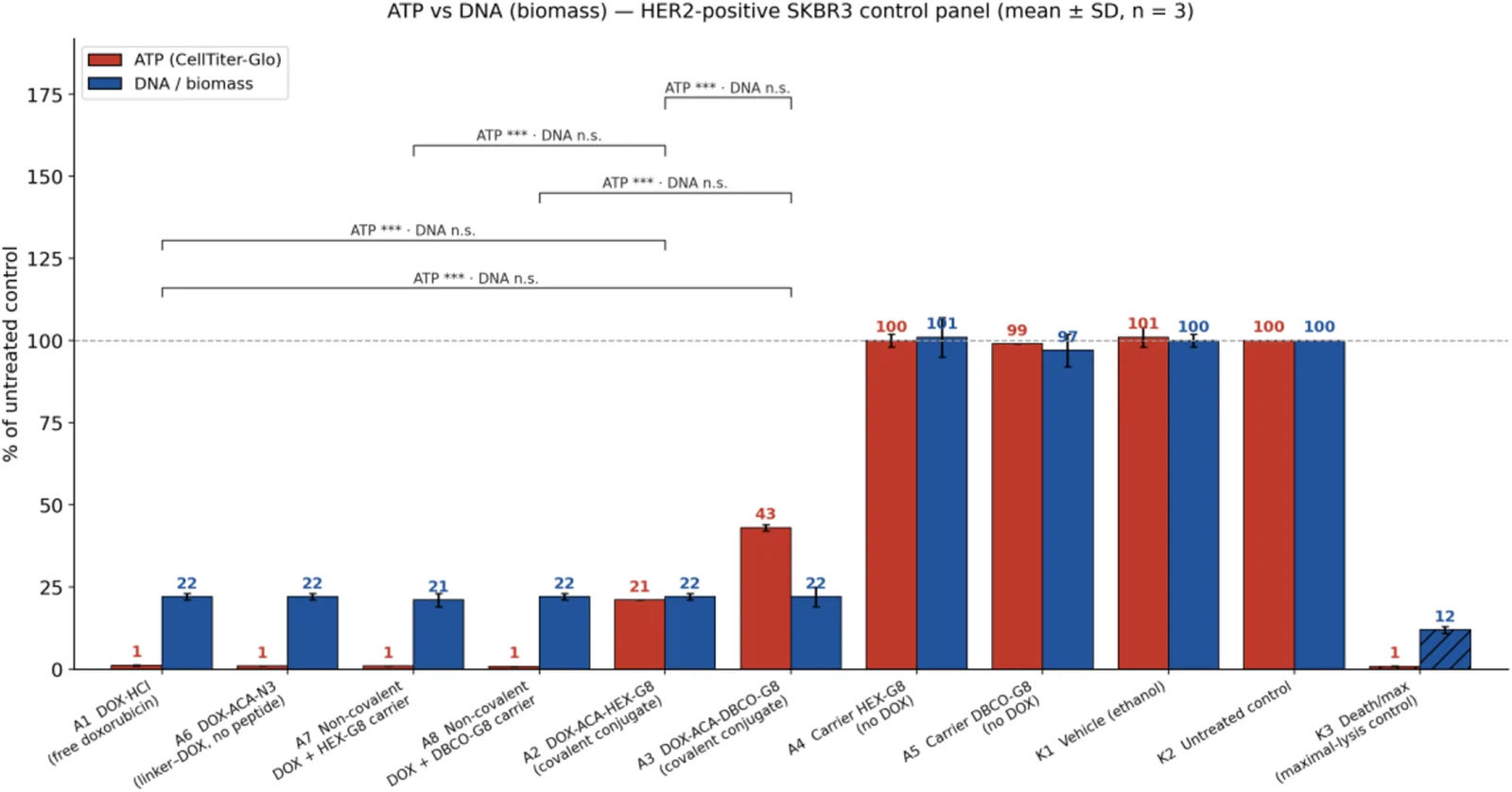
ATP versus DNA (biomass) on HER2-positive SKBR3 cells (10 μM, 72 h; mean ± SD, n = 3 independent experiments), each normalized to untreated control. The cytotoxic arms (free DOX, DOX-ACA-N_3_, mixtures) fall to ∼1% (ATP) and ∼22% (DNA) and the carriers match the control. Both covalent conjugates reduce both read-outs below control (ATP 21% for DOX-ACA-HEX-G8 and 43% for DOX-ACA-DBCO-G8; DNA/biomass ∼22%), with no hormesis, confirming a genuine loss of biomass by an orthogonal measure rather than an ATP-only effect. K2 (untreated) is the 100% reference; K3 (death/max) marks the floor of both read-outs (ATP ∼1%, DNA ∼12%). Brackets show Games-Howell *post hoc* comparisons on the per-experiment means (***p < 0.001; **p < 0.01; *p < 0.05; n. s., p > 0.05); exact p values for every contrast are given in Supplementary Table S11.

We then ran the identical control panel on HER2-positive SKBR3 cells (10 μM, 72 h; three independent experiments; Figures 6, 7; Table 7; raw data in Supplementary Tables S8–S10), where the picture was inverted relative to the non-malignant panel. Both covalent conjugates were cytotoxic: DOX-ACA-HEX-G8 reduced viability to ∼21% (ATP) and ∼22% (DNA/biomass) of control, and DOX-ACA-DBCO-G8 to ∼43% (ATP) and ∼22% (DNA/biomass). Biomass fell well below control for both conjugates (DNA ∼22%), with no ATP hormesis, so the conjugates suppress HER2-positive cells by an orthogonal biomass read-out and not by ATP alone. Because no t = 0 baseline was measured, this quantifies net biomass accumulated relative to a proliferating control and does not separate cell killing from growth arrest; “biomass” is used throughout in this operational sense.

**TABLE 7:** Control panel on HER2-positive SKBR3 cells (single concentration 10 μM, 72 h; mean ± SD of three independent experiments, three technical replicates each). Values are normalized within each experiment: LDH to that experiment’s maximal-lysis control K3 (sample/maximum × 100, without subtraction of the spontaneous signal), ATP and DNA to that experiment’s untreated control K2. Arms are listed in the order A1–A8, K1–K3. Entries marked “(ref.)” are the normalization reference for that read-out and are 100% by construction, with no dispersion. Every mean and SD was recomputed from the per-replicate values of Supplementary Tables S8–S10 by the procedure of Sections 2.7 and 2.9.

Arm	Compound	LDH (% of max)	ATP (% ctrl)	DNA (% ctrl)
A1	DOX·HCl (free doxorubicin)	100 ± 3	1.1 ± 0.1	22 ± 1
A2	DOX-ACA-HEX-G8 (covalent conjugate)	17 ± 2	21 ± 0	22 ± 1
A3	DOX-ACA-DBCO-G8 (covalent conjugate)	17 ± 1	43 ± 1	22 ± 3
A4	Carrier HEX-G8 (no DOX)	2.6 ± 0.4	100 ± 2	101 ± 6
A5	Carrier DBCO-G8 (no DOX)	3.3 ± 0.1	99 ± 0	97 ± 5
A6	DOX-ACA-N_3_ (linker–DOX, no peptide)	100 ± 5	0.9 ± 0.0	22 ± 1
A7	Non-covalent DOX + HEX-G8 carrier	99 ± 4	0.9 ± 0.0	21 ± 2
A8	Non-covalent DOX + DBCO-G8 carrier	99 ± 4	0.8 ± 0.0	22 ± 1
K1	Vehicle (ethanol)	3.0 ± 0.1	101 ± 3	100 ± 2
K2	Untreated control	3.0 ± 0.2	100 (ref.)	100 (ref.)
K3	Maximal-lysis control (death/max)	100 (ref.)	0.8 ± 0.1	11.9 ± 1.0

On HER2-positive cells the conjugates are cytotoxic (ATP 21% for DOX-ACA-HEX-G8 and 43% for DOX-ACA-DBCO-G8; DNA ∼22% of control) yet release little LDH (∼17%), i.e. they reduce biomass with minimal membrane lysis (non-lytic); free DOX and the mixtures are lytic (LDH ∼100%). Contrast Table 6 (non-malignant AC16), where the conjugates preserve biomass (DNA ∼90–94%) with low LDH, despite a partial ATP reduction.

Notably, the two read-outs no longer tracked each other for the DBCO conjugate: residual ATP exceeded the biomass loss (DOX-ACA-DBCO-G8, ATP ∼43% vs DNA ∼22%; ATP/DNA ≈ 1.9), whereas for DOX-ACA-HEX-G8 ATP and biomass agreed (∼21% vs ∼22%). ATP-based viability, therefore, overestimated survival for the DBCO conjugate; the retained ATP, together with low LDH, is consistent with a non-lytic outcome in which membrane integrity and part of the metabolic capacity persist while biomass is lost, and it reinforces the use of an orthogonal biomass read-out rather than ATP alone to quantify killing.

Free DOX, DOX-ACA-N_3_, and the non-covalent mixtures were likewise cytotoxic (ATP ∼1%, DNA ∼22% of control), while the carriers remained inert (∼100%). Crucially, the two conjugates released little LDH (∼17% each) despite reducing biomass to ∼22% of the proliferating control, whereas free DOX and the mixtures gave near-maximal LDH (∼100%). The conjugates therefore suppress SKBR3 with far less membrane rupture than free DOX, which is consistent with a non-lytic mode of action. Whether that mode reflects apoptosis or cytostasis cannot be decided here and requires caspase-3/7 and annexin-V assays. Read together with the non-malignant panel, a single orthogonal (DNA) read-out captures the selectivity of the lead compound: DOX-ACA-HEX-G8 lowered SKBR3 biomass to ∼22% of control while leaving AC16 cardiomyocyte biomass at ∼94%.

The arms differed by Welch one-way ANOVA (p < 0.001 for ATP, DNA, and LDH). On tumor-cell biomass the conjugates were indistinguishable from free DOX and from the non-covalent mixtures: DNA was 21%–22% of control in every DOX-bearing arm, with Games-Howell p = 1.0 for every pairwise contrast among them. Against SKBR3 biomass the arms are therefore equally effective. They diverged sharply, however, on membrane lysis (LDH ∼17% versus ∼100%, a difference of ∼83 percentage points; Games-Howell p ≤ 0.003) and on residual ATP (21% and 43% versus ∼1%; p ≤ 0.001), consistent with an equal reduction of biomass achieved through a different, non-lytic route. Statistics are Welch one-way ANOVA with Games-Howell *post hoc* tests on per-experiment means (n = 3 independent experiments, three technical replicates each). With n = 3 the *post hoc* p values are unstable and are quoted only to indicate which contrasts are supported; the effect sizes carry the argument. Exact p values for every contrast quoted are given in Supplementary Table S11. Full per-replicate values are in Supplementary Tables S8–S10.

### 3.9 Neither the doxorubicin chromophore nor the guanidinium carrier interferes with the luminescence read-out

**FIGURE 8:**
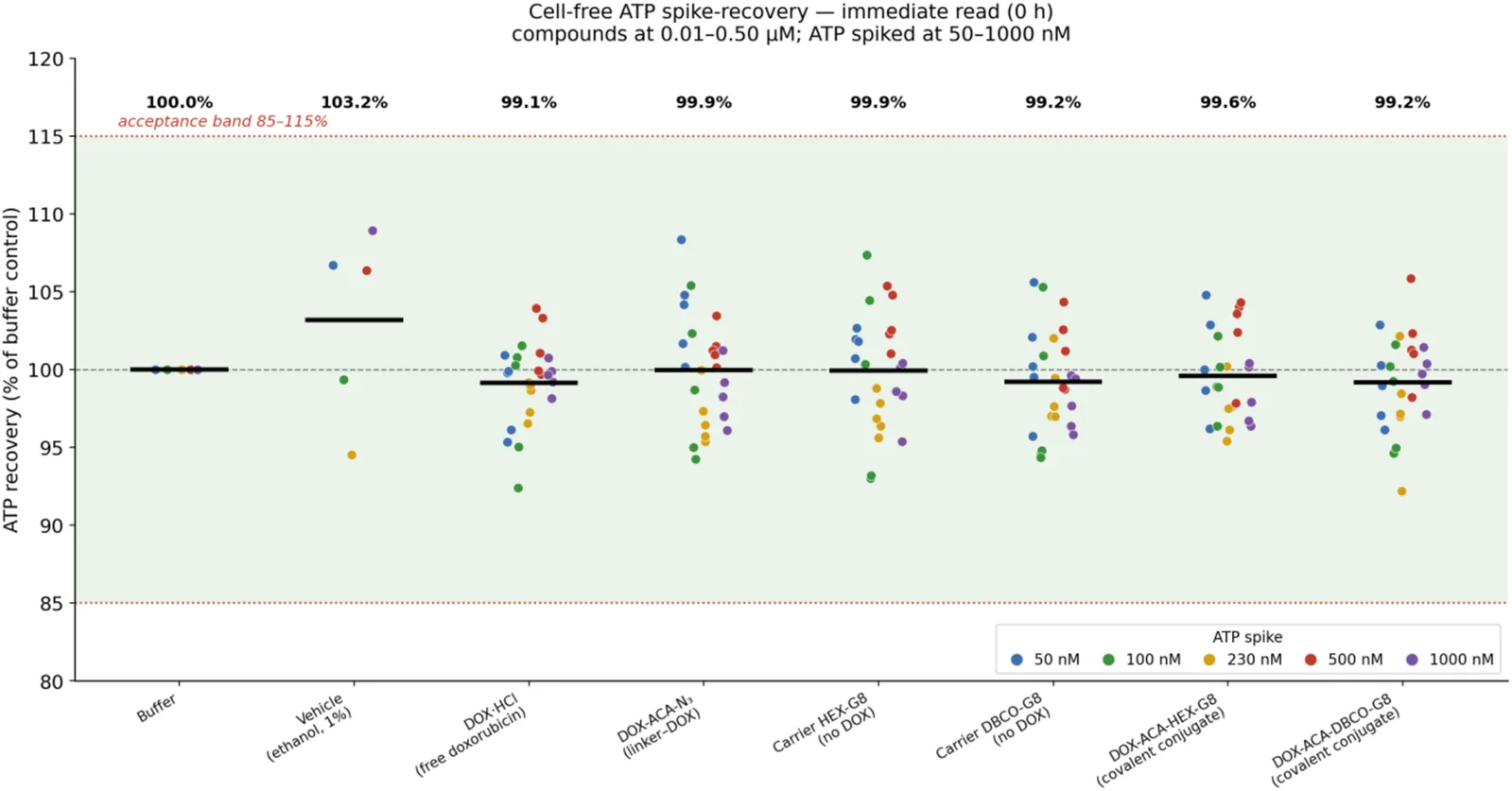
Cell-free ATP spike-recovery control. Recovery of a known ATP spike measured in the absence of cells, in the medium and reagent used for the AC16 spheroid experiments, in a white 96-well flat-bottom plate, and read immediately, reproducing the interval over which ATP and test article are in contact in the cell-based assay. Each point is one determination; color denotes the ATP spike level (50, 100, 230, 500 or 1,000 nM) and the horizontal bar is the mean of the arm across all compound concentrations and spike levels. Recovery is the background-corrected signal of the arm divided by the background-corrected signal of the buffer arm at the same spike level; a compound-only blank was subtracted for every arm and concentration. Test articles were assayed at 0.01–0.50 µM and ethanol was held at 1% (v/v) in every well. The shaded band and the dotted lines mark the 85%–115% acceptance criterion. No determination fell outside the band (range 92.2%–108.9%, median 99.6%, n = 155 recovery values; the five buffer wells define 100% and are plotted for reference, 160 wells in total). Recovery was independent of the spike level and did not differ between arms containing the DAPEG carrier and those without it (p = 0.49). The ethanol vehicle gave the largest deviation of any arm (mean 103.2%). Raw per-well data are given in Supplementary Table S12.

The spike-recovery design comprised 160 wells (Figure 8; Supplementary Table S12): buffer and vehicle references, and six test articles each assayed at five compound concentrations, all across five ATP spike levels. Across the 155 recovery values (all wells except the five buffer references, which are the denominator), recovery ranged from 92.2% to 108.9% of the buffer control, with a median of 99.6%, and none fell outside the 85%–115% band. Mean recovery was 99.1% for free DOX, 99.9% for DOX-ACA-N_3_, 99.9% and 99.2% for the bare HEX-G8 and DBCO-G8 carriers, and 99.6% and 99.2% for DOX-ACA-HEX-G8 and DOX-ACA-DBCO-G8. Recovery was independent of the spike level, and the arms containing the DAPEG carrier did not differ from those without it (99.5% versus 99.9%; p = 0.49), so there is evidence neither of a multiplicative deficit nor of saturable binding of ATP by the polycationic carrier.

The ethanol vehicle was the one arm that deviated. At 1% (v/v) it gave a mean recovery of 103.2% with the widest dispersion of any arm, reaching 108.9% at the highest spike level. The effect is small and cannot account for an elevation to 278% of control, but it indicates that the vehicle contributes a few percentage points to the ATP signal even in a cell-free system.

The signal elevation reported in Section 3.5 is therefore not an artifact of the detection chemistry. At the concentrations at which it occurs, and over the interval during which ATP and test article are in contact in the assay, the conversion of ATP to luminescence is unaffected by the doxorubicin chromophore, by the guanidinium carrier, or by the conjugates.

## 4 Discussion

The central finding of this study is a dissociation between potency and selectivity. Free doxorubicin is exquisitely potent against HER2-positive spheroids but is equally lethal to cardiomyocytes and normal breast epithelium, so it offers essentially no *in vitro* therapeutic window, an observation that mirrors the clinical reality of anthracycline cardiotoxicity. DOX-ACA-HEX-G8 reproduces DOX-level potency against SKBR3 at 48–72 h while being 15-fold (HB2) and more than 18-fold (AC16) less toxic to the non-malignant lines than free DOX. The separation is visible without any extrapolation: at 0.5 µM/72 h free DOX leaves cardiomyocytes at 14% of control and SKBR3 at 6%, whereas DOX-ACA-HEX-G8 leaves cardiomyocytes at 122% and SKBR3 at 32% (Table 4). If confirmed, this is the most translationally meaningful result of the project, because it addresses the specific liability, cardiac injury, that limits DOX in the clinic. The DOX-ACA-DBCO-G8 comparator, identical in guanidinium valency but built on a different linker, is markedly less selective, showing that the effect tracks with linker chemistry and is not a generic consequence of guanidinium-rich conjugation.

Spheroids impose diffusion barriers, cell–cell contact, and anti-apoptotic signaling that raise DOX IC_50_ values relative to matched monolayers; multiple studies report several-fold higher IC_50_ in 3D (Carbone et al., 2025; Imamura et al., 2015), and MDA-MB-231 in particular forms loose spheroids whose behavior is intermediate. At the diameters measured here (131–154 μm at 24 h, 297–321 μm at 48 h, and 333–367 μm at 72 h; Section 2.4) an oxygen-limited core cannot be excluded from 48 h onward, so restricted penetration, 3D architecture and a possible hypoxic core all remain candidate contributors and are not separated by these data. A large peptidomimetic–DOX conjugate is also expected to penetrate a spheroid more slowly than free DOX, which compounds this effect. Consistent with this, doxorubicin autofluorescence imaging showed that both conjugates are internalized (Figure 3), so the lower potency is not due to failed uptake but more plausibly to slower penetration and/or limited intracellular drug release.

Why the benefit is restricted to SKBR3 is an important open question, taken up below together with the release mechanism; quantitative uptake and a defined drug-release assay are the logical next experiments.

The hormetic signal deserves particular caution. Apparent viabilities of 200%–280% of control in an ATP-based luminescence assay can arise from genuine low-dose proliferative or metabolic stimulation (Calabrese and Baldwin, 2003). A precedent exists within our own work. Using the same DAPEG scaffold, the same nonadherent spheroid protocol and the same CellTiter-Glo 3D read-out—but in a different cell line (HEK-293T) and with N-acylated (lipidated) rather than click-conjugated analogs—we previously observed a biphasic, wave-like viability profile, with growth stimulation at low and at high concentrations and a minimum near 5–10 µM. Some analogs raised the apparent cell number several-fold above untreated controls (Mallek et al., 2025). That study also reported growth stimulation by MTT in monolayers, i.e. by a read-out that does not depend on ATP or luciferase, which argues that the phenomenon is at least partly a property of the carrier rather than of the luminescence chemistry. That earlier study also reported that the carriers themselves stimulate growth, which is not reproduced by the single 10 µM point at which the bare carriers are inert here (Tables 6 and 7); a carrier dose–response across the full range is required before the elevation can be attributed to the carrier, to the conjugate, or to the read-out.

The vehicle itself is a candidate that the present design largely excludes. Ethanol is a documented hormetic agent: at 0.005%–1% (v/v), it roughly doubles fibroblast tetrazolium reduction activity, reaching ∼198% of control at 0.1%, and cell counting confirms that this reflects metabolic stimulation rather than proliferation (Kar et al., 2021). The magnitude, the direction and the metabolic-not-proliferative signature all match what we observe. Three considerations nonetheless argue against attributing our elevation to the solvent. First, the read-out differs: in that study ethanol raised the tetrazolium signal about two-fold while ATP stayed at 102% ± 11% of control at 0.01% and fell to 81% ± 5% at 1% and 77% ± 16% at 3%, so the ethanol-driven elevation reported in that study is largely confined to the oxidoreductase read-out. Our own cell-free measurement qualifies this rather than confirming it: at 1% (v/v) the vehicle raised the ATP signal by a few percentage points, with the largest deviation of any arm (Section 3.9). The contribution is far too small to account for an elevation to 278% of control, and it cannot generate a compound-dependent difference, but we do not claim that the ATP read-out is insensitive to the vehicle. Second, the vehicle is constant: every well, including the vehicle control, received the same 1% (v/v), so a solvent effect cannot produce an elevation that depends on the compound present. Third, and decisively, free DOX in the same vehicle leaves AC16 at 117% of control at 0.5 µM/48 h whereas DOX-ACA-DBCO-G8 leaves it at 278% (Table 5).

One caveat survives this argument and is not resolved by it. A constant solvent concentration is not necessarily a constant offset: ethanol increases membrane fluidity and disorders the lipid bilayer, and disorganizes the actin cytoskeleton from ∼0.1 to 1% upward (Kar et al., 2021), and these are the same membrane interactions through which guanidinium-rich transporters enter cells.

Separately, the elevation can reflect assay interference: guanidinium-rich peptides can perturb membrane integrity and cellular ATP handling, and both DOX and the conjugates have optical properties that can affect luminescence read-outs. The divergence we observe between ATP and biomass is itself expected rather than anomalous: hormesis is endpoint-specific, and a dose that is stimulatory for one property can be inhibitory for another, as ethanol is for tetrazolium reduction and mitochondrial integrity at the same 1% (Kar et al., 2021). That an ATP signal rises while DNA does not is therefore not evidence that the ATP signal is artifactual; it locates the effect in metabolism rather than in cell number, and leaves its cause open. The cell-free control reported in Section 3.9 removes one of these two possibilities. Recovery of a known ATP spike was complete in the presence of every test article, across the concentration range in which the elevation is seen and over the interval during which ATP and compound are in contact in the assay, so the elevation is not produced by the detection chemistry. What remains is a cellular effect whose mechanism is open: at low concentrations the conjugates raise the ATP content of non-malignant spheroids without raising their biomass, which locates the effect in metabolism rather than in cell number. IC_50_ values fitted across the elevated region remain provisional, since a biphasic response still violates the assumptions of the inhibition model. This directly affects the selectivity estimates that constitute the paper’s main claim. Moreover, low-dose metabolic stimulation of cardiomyocytes is not necessarily benign: guanidinium-rich agents can elicit stress-adaptive metabolic responses, so the hormetic elevation warrants functional follow-up of cardiomyocytes rather than being treated as assay noise.

The control panel for non-malignant cells strengthens the causal interpretation of the selectivity but does not clarify the hormetic effect. A non-covalent DOX + carrier mixture is as cytotoxic as free DOX, whereas the covalent conjugate is not. The reduced toxicity toward non-malignant cells is therefore a genuine property of the covalent construct rather than an artifact of co-administration, and it is conferred by the guanidinium peptidomimetic and not the linker. It should not, however, be equated with the cells being unaffected. Biomass preservation and low membrane lysis coexist with a substantial loss of ATP for the lead compound (DOX-ACA-HEX-G8, ∼44% of control): cardiomyocyte spheroids retain their number and membrane integrity while operating at roughly half the metabolic signal of untreated controls. On cardiomyocytes taken in isolation, the lead is in fact the more aggressive of the two conjugates—twice the LDH release and half the residual ATP of DOX-ACA-DBCO-G8 at 10 μM, and a 72 h IC_50_ of 8.92 versus 19.7 µM—so the term “sparing” describes its position relative to free doxorubicin, not relative to its comparator.

Because mitochondrial injury is central to clinical anthracycline cardiotoxicity, this divergence is a finding in its own right rather than a nuisance, and it cannot be resolved with viability assays alone; mitochondrial membrane potential, oxygen consumption and contractile function are the appropriate next read-outs. At 10 µM/72 h, however, the conjugates did not elevate ATP: ATP fell to ∼44% (DOX-ACA-HEX-G8) and ∼82% (DOX-ACA-DBCO-G8) of control while biomass was largely preserved (DNA ∼90–94%), so ATP sat at or below biomass rather than above it and this single high dose therefore does not characterize the low-dose elevation, which is addressed instead by the cell-free control of Section 3.9.

Extending the control panel to HER2-positive SKBR3 cells completed the selectivity argument within a single, internally consistent framework. On cancer cells, both conjugates genuinely reduced biomass by an orthogonal read-out (DNA, not ATP alone) and, unlike free DOX, did so with little LDH release, pointing to a non-lytic mode of action. The same lead compound that reduced SKBR3 biomass to ∼22% of control left cardiomyocyte biomass essentially intact (∼94%). Covalent guanidinium-peptidomimetic conjugation, therefore, appears to do two things at once: it preserves activity against HER2-positive cells while shifting the route away from membrane lysis, and it spares non-malignant cells, including cardiomyocytes. A caspase-3/7 assay and an LDH/annexin time course would establish whether the non-lytic route is apoptosis or cytostasis.

Two aspects of the biology remain to be explained and are, at this stage, hypotheses rather than results. First, why is the benefit confined to the HER2-positive model? Guanidinium-rich transporters enter cells through interactions with anionic cell-surface components (heparan-sulfate proteoglycans and phospholipids) and through endocytic routes, all of which differ between breast-cancer subtypes; HER2-amplified cells are also highly endocytically active. Differences in surface charge, glycocalyx composition, endocytic flux, and ABC-transporter (efflux) expression between SKBR3 and MDA-MB-231 could modulate conjugate uptake, trafficking, and retention, and any of these, rather than HER2 signaling *per se*, may underlie the subtype difference. Because only one line per subtype was tested, this association must not be over-interpreted; target-engagement experiments and a broader cell-line panel are required.

Second, how the conjugate exerts anthracycline pharmacology. The 1,2,3-triazole formed by CuAAC/SPAAC is metabolically stable and is not a designed cleavage site, so doxorubicin is unlikely to be liberated at the triazole itself. Activity could instead arise from lysosomal or esterase/protease-mediated cleavage of the more labile bonds of the aminocaproyl (ACA) spacer, releasing free doxorubicin intracellularly (Lelle et al., 2014; Vrettos et al., 2018), or, less conventionally, from a still-conjugated species reaching the nucleus. The shift from a lytic (free DOX) to an apparently non-lytic (conjugate) mode in SKBR3 cells is consistent with slower, compartmentalized intracellular release. A defined drug-release assay and nuclear localization imaging are needed to distinguish among these possibilities.

These findings should be read against the current state of the field. HER2-selective cell-penetrating systems that deliver doxorubicin, spare non-target cells, and remain active in 3D spheroids have already been reported (Chong et al., 2021). The incremental contribution here is therefore the explicit cardiomyocyte counter-screen and the linker dependence of selectivity, rather than cell-penetrating delivery *per se*. For peptide–doxorubicin conjugates, endosomal entrapment and limited intracellular release are recognized as the principal efficiency bottleneck (Grabeck et al., 2022; Vale et al., 2020); because doxorubicin release from the non-cleavable triazole was not measured here, whether the conjugate acts intact or liberates free drug remains the decisive open parameter for both potency and selectivity.

## 5 Strengths and limitations

### 5.1 Strengths

The main strength of this study is a cell panel that enables a genuine therapeutic window readout. Two molecular subtypes (HER2-positive SKBR3, triple-negative MDA-MB-231) are paired with two non-malignant counterparts, including AC16 cardiomyocytes, which most conjugate studies omit. Viability was measured in 3D CellTiter-Glo spheroids rather than in monolayers, a model that better reproduces the diffusion barriers and cell–cell contacts of a solid tumor. The analysis is restricted to the two eight-guanidinium conjugates. The linker-matched pair (hexynoyl versus DBCO) isolates a single design axis, and DOX-ACA-DBCO-G8 serves as an internal control indicating linker-dependent selectivity. Doxorubicin autofluorescence confirms internalization, a cell-free spike-recovery control establishes that the luminescence read-out is not distorted by any of the test articles, and IC_50_ values were obtained by four-parameter logistic regression with bootstrap confidence intervals and free asymptotes to accommodate the low-dose hormesis.

### 5.2 Limitations and risks

The conclusions are bounded by several limitations. Each subtype rests on a single line, and the benefit is confined to the HER2-positive model, since both conjugates are less potent than free doxorubicin against MDA-MB-231. A determinable selectivity index for free doxorubicin is not obtainable from these data, because its 72 h IC_50_ is censored on both lines of the relevant ratio—below the lowest tested concentration on SKBR3 and on AC16 alike. Establishing the size of the window in IC_50_ terms therefore requires sub-micromolar dose–response on both cell lines, not on the tumor line alone; until that is available the reduced toxicity is quantified by the two censoring-free measures of Tables 3 and 4. The mechanism is not established, because doxorubicin release from the non-cleavable triazole was not observed. The LDH/ATP/DNA panels show that covalent conjugation is required to spare non-malignant cells and that the conjugates reduce tumor-cell biomass with reduced membrane lysis (a non-lytic mode), but discriminating apoptosis from cytostasis requires a caspase/annexin time course and a t = 0 baseline.

The low-dose hormesis is assigned a metabolic rather than proliferative basis: the DNA read-out does not rise with ATP, and the cell-free control of Section 3.9 excludes an artifact of the detection chemistry. Its mechanism is nevertheless unresolved. One control bearing on that assignment remains absent: a dose–response for the unconjugated carriers across the full concentration range. The carriers were tested at a single concentration (10 µM), at which they are inert in both cell lines; their behaviour in the low-dose region where the elevation occurs was not measured, so the elevation cannot yet be attributed to the carrier, to the conjugate, or to the read-out. The 72 h viability read-out shows absence of acute cardiomyocyte cytotoxicity, but it does not probe the topoisomerase-2β, mitochondrial, and mtDNA–cGAS–STING mechanisms of clinical anthracycline cardiotoxicity (Lei et al., 2023; Xiong et al., 2025; Zhang et al., 2012), so sparing is not cardioprotection. This proof of concept is limited to the two eight-guanidinium conjugates.

A further constraint follows from the model itself. AC16 was derived by fusion of primary adult human ventricular cells with SV40-transformed fibroblasts, and was characterized at generation as being at a precontractile developmental stage, with myofibrils confined to the subsarcolemmal region (Davidson et al., 2005). A subsequent systematic comparison against primary and human iPSC-derived cardiomyocytes reported low expression of sarcomeric α-actinin, troponin I and caveolin-3, and low resemblance to mature adult cardiac tissue irrespective of the differentiation protocol applied (Onódi et al., 2022). Contractile read-outs are therefore not available in this system and would require a contractile model; mitochondrial membrane potential (JC-1 or TMRM) is feasible in AC16 spheroids and is the immediate next functional measurement indicated by the ATP–biomass divergence reported here.

## 6 Conclusion

In a physiologically relevant 3D spheroid panel, guanidinium-rich peptidomimetic conjugation did not increase the raw potency of doxorubicin. However, the eight-guanidinium hexynoyl conjugate DOX-ACA-HEX-G8 decoupled anticancer activity from cardiomyocyte and normal-epithelium toxicity in a HER2-positive model (SKBR3). It is 15-fold (HB2) and more than 18-fold (AC16) less toxic than free doxorubicin to the non-malignant lines, while matching it against SKBR3 at 48–72 h, and at 0.5 µM/72 h, it separates cardiomyocytes from tumor cells by 90 percentage points of viability, where free doxorubicin separates them by 8. Because the SKBR3 IC_50_ is censored for both compounds, their selectivity indices are lower bounds (>18 and >23 for the conjugate) and the fold-change between them is not determinable; the size of the window in IC_50_ terms therefore remains to be established by sub-micromolar dosing. Doxorubicin autofluorescence confirmed that the conjugates are internalized. The linker-matched comparator DOX-ACA-DBCO-G8 was less selective, showing the effect is linker-dependent. The benefit did not extend to the triple-negative model. A pronounced low-dose hormetic signal in the non-malignant lines is not an artifact of the read-out, since recovery of a known ATP spike was complete in the presence of every test article in a cell-free control, and is not proliferation, since biomass does not rise with ATP; its mechanism remains open. DOX-ACA-HEX-G8 is a credible lead for anthracycline delivery with reduced acute cardiomyocyte toxicity, contingent on confirmation with orthogonal viability read-outs, an expanded control arm, independent biological replication, and mechanistic uptake/release studies.

## Data Availability

The data presented in the study are deposited in the figshare repository, accession number 10.6084/m9.figshare.33058754.
