# Supplementary material for: Guanidinium-rich cell-penetrating peptidomimetic–doxorubicin conjugates show linker-dependent, reduced acute toxicity to cardiomyocytes and normal breast epithelium while retaining activity against a HER2-positive breast cancer model (SKBR3 spheroids)
Source: Front Chem. 2026 Sep 10;14:1945333. doi: 10.3389/fchem.2026.1945333 (PMC13601302; doi:10.3389/fchem.2026.1945333)
Supplement: Supplementary file 1 [file DataSheet1.docx]

**Supplementary Material**

**S1. Supplementary Methods**

**S1.1 Peptidomimetic synthesis and conjugation**

Peptidomimetics were assembled by solid-phase synthesis using orthogonally protected 2,3-diaminopropionic acid (Dap) building blocks with poly(ethylene glycol)-type O2Oc spacers at the N- and C-termini. Dap side chains were guanidinylated (GO2 group) to give the eight-guanidinium scaffold, and selective deprotection was monitored by the chloranil and Kaiser tests. An alkyne-terminated hexynoyl linker (for DOX-ACA-HEX-G8) or a dibenzocyclooctyne (DBCO) linker (for DOX-ACA-DBCO-G8) was introduced at the N-terminus. Doxorubicin was functionalized with an azide linker (DOX-ACA-N3). The two conjugates were then formed by complementary click chemistry: DOX-ACA-HEX-G8 by copper-catalyzed azide–alkyne cycloaddition (CuAAC; sodium ascorbate/CuSO4 in H2O:EtOH with THF/DMF, 25 °C, 24 h, protected from light) and DOX-ACA-DBCO-G8 by strain-promoted azide–alkyne cycloaddition (SPAAC; PBS:THF, 25 °C, 24 h). Products were lyophilized and purified from excess reagents by dialysis (Slide-A-Lyzer G3 cassettes, 2 kDa MWCO; 0.25% aqueous acetic acid).

**S1.2 Three-dimensional (3D) spheroid CellTiter-Glo assay**

Spheroids of MDA-MB-231, SKBR3, HB2, and AC16 were treated with 0.5, 1, 2, 5, 10, 20, and 50 µM. The 50 µM point is present for HB2 and AC16 at all three time points, for all four lines at 24 h, and for both conjugates on SKBR3 at 48 and 72 h; it was not acquired at 48 and 72 h for MDA-MB-231 (any compound) or for free DOX on SKBR3, where the tables report n.d. compound for 24, 48, and 72 h and assayed with CellTiter-Glo 3D (ATP-dependent luminescence). Raw luminescence was normalized to the untreated control (100%). Four-parameter logistic regression with free asymptotes and bootstrap 95% CIs (300 iterations) was used to estimate IC50 (main text, Section 2.5).

**S2. Normalized viability (mean ± SD, % of control)**

*Tables S1–S4 give the underlying CellTiter-Glo 3D viabilities used for curve fitting (mean ± SD of technical replicates). Values above 100% at low concentrations reflect the hormetic response discussed in the main text.*

*Table S1. MDA-MB-231 normalized viability (% of control); columns are compound × time point.*

| **µM** | **DOX-HCl 24h** | **DOX-HCl 48h** | **DOX-HCl 72h** | **DOX-ACA-HEX-G8 24h** | **DOX-ACA-HEX-G8 48h** | **DOX-ACA-HEX-G8 72h** | **DOX-ACA-DBCO-G8 24h** | **DOX-ACA-DBCO-G8 48h** | **DOX-ACA-DBCO-G8 72h** |
| --- | --- | --- | --- | --- | --- | --- | --- | --- | --- |
| 0 | 100±14 | 100±1 | 100±4 | 100±14 | 100±16 | 100±19 | 100±14 | 100±16 | 100±19 |
| 0.5 | 89±19 | 75±8 | 75±9 | 90±17 | 83±10 | 91±9 | 92±9 | 102±24 | 80±23 |
| 1 | 79±7 | 52±16 | 44±3 | 106±14 | 85±13 | 85±7 | 92±10 | 95±14 | 71±18 |
| 2 | 70±11 | 37±20 | 12±1 | 105±9 | 89±11 | 93±15 | 99±8 | 82±10 | 66±17 |
| 5 | 66±11 | 28±15 | 4±1 | 99±7 | 73±37 | 102±10 | 96±11 | 75±11 | 66±13 |
| 10 | 70±8 | 26±17 | 5±3 | 75±8 | 104±9 | 116±14 | 97±6 | 74±20 | 55±6 |
| 20 | 71±1 | 19±11 | 4±2 | 37±6 | 110±12 | 120±19 | 98±3 | 58±15 | 40±7 |
| 50 | 54±8 | n.d.ᵃ | n.d.ᵃ | 10±5 | n.d.ᵃ | n.d.ᵃ | 78±11 | n.d.ᵃ | n.d.ᵃ |

*Values are mean ± SD of the technical replicate wells of a single plate per time point (four to six wells per concentration). Each plate was normalized to its own control wells: 24 h, A8–A12 and B12–H12 (n = 12); 48 h and 72 h conjugate plates, G1–G5 and H1–H5 (n = 10); 48 h DOX plate, F6–F7 (n = 2; the column-12 wells were excluded because of a plate-edge effect); 72 h DOX plate, F12–H12 and G11–H11 (n = 5). At 24 h all three test articles were on one plate; at 48 and 72 h the conjugates and free DOX were on separate plates, whose controls differ (72 h: 87 564 versus 60 510 RLU), so cross-plate normalization is not valid. On the 48 h conjugate plate the whole of row C was excluded because of a documented pipetting failure; the criterion was applied to the entire row and to both conjugates, irrespective of the values obtained. ᵃ n.d., not determined: the 50 µM point was not tested at 48 and 72 h. Two single-well failures were excluded. On the 72 h DOX plate, one well (row D, 10 µM) read 125% of control while the other four wells of that group read 1–7%; the reading is at the level of an untreated control, consistent with the compound not having been dispensed. On the 72 h conjugate plate, one DOX-ACA-HEX-G8 well (row B, 20 µM) read 40% of control while the other four read 97–136%. In both cases the remaining wells of the affected row were indistinguishable from their neighbors at every other concentration and were retained. Excluding the second well raises the reported value from 104% to 120% of control, i.e. away from rather than toward the interpretation advanced in the text.*

*Table S2. SKBR3 normalized viability (% of control); columns are compound × time point.*

| **µM** | **DOX-HCl 24h** | **DOX-HCl 48h** | **DOX-HCl 72h** | **DOX-ACA-HEX-G8 24h** | **DOX-ACA-HEX-G8 48h** | **DOX-ACA-HEX-G8 72h** | **DOX-ACA-DBCO-G8 24h** | **DOX-ACA-DBCO-G8 48h** | **DOX-ACA-DBCO-G8 72h** |
| --- | --- | --- | --- | --- | --- | --- | --- | --- | --- |
| 0 | 100±8 | 100±3 | 100±4 | 100±8 | 100±3 | 100±4 | 100±8 | 100±3 | 100±4 |
| 0.5 | 66±3 | 16±1 | 6±0 | 92±8 | 27±4 | 32±6 | 81±4 | 84±5 | 76±6 |
| 1 | 61±3 | 8±1 | 5±0 | 93±9 | 34±3 | 46±3 | 79±4 | 88±11 | 75±7 |
| 2 | 32±9 | 10±2 | 8±1 | 82±4 | 32±10 | 42±2 | 84±9 | 85±5 | 72±9 |
| 5 | 16±1 | 13±3 | 10±1 | 69±4 | 34±14 | 37±2 | 76±1 | 77±10 | 62±4 |
| 10 | 17±0 | 15±4 | 9±1 | 60±4 | 45±2 | 26±2 | 67±1 | 68±9 | 52±6 |
| 20 | 25±1 | 2±1 | 1±0 | 39±7 | 22±8 | 23±3 | 64±1 | 51±29 | 42±1 |
| 50 | 15±3 | n.d. | n.d. | 12±6 | 4±6 | 17±6 | 54±8 | 20±18 | 32±6 |

*Table S3. HB2 normalized viability (% of control); columns are compound × time point.*

| **µM** | **DOX-HCl 24h** | **DOX-HCl 48h** | **DOX-HCl 72h** | **DOX-ACA-HEX-G8 24h** | **DOX-ACA-HEX-G8 48h** | **DOX-ACA-HEX-G8 72h** | **DOX-ACA-DBCO-G8 24h** | **DOX-ACA-DBCO-G8 48h** | **DOX-ACA-DBCO-G8 72h** |
| --- | --- | --- | --- | --- | --- | --- | --- | --- | --- |
| 0 | 100±7 | 100±25 | 100±10 | 100±7 | 100±25 | 100±10 | 100±7 | 100±25 | 100±10 |
| 0.5 | 122±11 | 93±6 | 66±9 | 180±10 | 203±11 | 166±57 | 186±9 | 210±16 | 141±22 |
| 1 | 96±9 | 97±22 | 45±5 | 159±9 | 164±15 | 122±14 | 196±24 | 180±14 | 121±9 |
| 2 | 113±13 | 38±10 | 9±1 | 155±11 | 165±7 | 101±19 | 181±14 | 190±24 | 114±17 |
| 5 | 90±10 | 13±5 | 4±3 | 145±16 | 132±14 | 83±18 | 165±16 | 151±20 | 101±11 |
| 10 | 105±17 | 16±1 | -0±0 | 134±11 | 111±16 | 54±10 | 146±13 | 134±16 | 80±9 |
| 20 | 82±15 | 12±5 | 2±1 | 87±12 | 69±11 | 37±7 | 123±8 | 116±11 | 73±3 |
| 50 | 51±3 | 1±0 | -1±0 | 32±9 | 12±5 | 1±1 | 104±17 | 65±8 | 27±4 |

*Table S4. AC16 normalized viability (% of control); columns are compound × time point.*

| **µM** | **DOX-HCl 24h** | **DOX-HCl 48h** | **DOX-HCl 72h** | **DOX-ACA-HEX-G8 24h** | **DOX-ACA-HEX-G8 48h** | **DOX-ACA-HEX-G8 72h** | **DOX-ACA-DBCO-G8 24h** | **DOX-ACA-DBCO-G8 48h** | **DOX-ACA-DBCO-G8 72h** |
| --- | --- | --- | --- | --- | --- | --- | --- | --- | --- |
| 0 | 100±7 | 100±11 | 100±16 | 100±7 | 100±11 | 100±16 | 100±7 | 100±11 | 100±16 |
| 0.5 | 84±2 | 117±18 | 14±2 | 107±8 | 115±27 | 122±14 | 107±7 | 278±12 | 121±15 |
| 1 | 73±5 | 85±6 | 7±1 | 102±1 | 117±16 | 102±2 | 103±5 | 275±12 | 136±5 |
| 2 | 56±4 | 54±7 | 1±0 | 91±1 | 102±26 | 94±6 | 94±2 | 220±11 | 140±2 |
| 5 | 50±1 | 17±3 | 2±1 | 79±6 | 71±18 | 69±10 | 102±8 | 196±5 | 115±5 |
| 10 | 47±5 | 22±8 | 3±0 | 68±6 | 49±11 | 46±3 | 90±1 | 133±13 | 80±2 |
| 20 | 42±6 | 16±3 | 2±1 | 46±3 | 28±9 | 24±0 | 64±2 | 68±3 | 55±6 |
| 50 | 24±5 | 8±4 | 1±0 | 20±2 | 4±2 | 1±0 | 29±5 | 19±2 | 7±1 |

**S3. Control-panel raw data and statistics**

*Arms were tested in three independent experiments (three technical replicates each) at 10 µM, 72 h. The complete per-value raw data for the AC16 and SKBR3 control panels are given below. Within-arm dispersion differs across arms by more than an order of magnitude, so the equal-variance assumption of classical ANOVA is not met and Welch one-way ANOVA with Games-Howell post-hoc tests was used throughout (per-experiment means, n = 3 independent experiments). Differences among arms were significant for every read-out (Welch ANOVA p < 0.001; Table S11). The pairwise contrasts are not uniformly significant at p < 0.001: on AC16 the conjugate-versus-DOX contrasts reach p = 0.001–0.02 depending on the read-out, and the two conjugates are indistinguishable on AC16 LDH and on biomass in both lines. Exact p values for every contrast quoted in the main text are given in Table S11. With n = 3 these p values are unstable and are reported only to indicate which contrasts the data support; the effect sizes carry the argument.*

*Table S5 . AC16 — ATP (raw) (E = experiment; nine technical values per arm).*

| **Arm** | **E1.1** | **E1.2** | **E1.3** | **E2.1** | **E2.2** | **E2.3** | **E3.1** | **E3.2** | **E3.3** |
| --- | --- | --- | --- | --- | --- | --- | --- | --- | --- |
| A1 DOX·HCl (free doxorubicin) | 1692 | 1643 | 1534 | 1428 | 1397 | 1488 | 1376 | 1291 | 1635 |
| A2 DOX-ACA-HEX-G8 (covalent conjugate) | 51204 | 54312 | 55180 | 52940 | 50865 | 53910 | 52440 | 56124 | 51060 |
| A3 DOX-ACA-DBCO-G8 (covalent conjugate) | 95840 | 93359 | 106945 | 96660 | 108334 | 101907 | 89089 | 92594 | 106155 |
| A4 Carrier HEX-G8 (no DOX) | 135621 | 144802 | 141012 | 128619 | 118543 | 123412 | 136006 | 45672 | 127112 |
| A5 Carrier DBCO-G8 (no DOX) | 145671 | 118637 | 120671 | 128412 | 135912 | 126009 | 117835 | 136183 | 107429 |
| A6 DOX-ACA-N3 (linker–DOX, no peptide) | 1287 | 1312 | 1652 | 1523 | 1637 | 1531 | 1734 | 1123 | 1542 |
| A7 Non-covalent DOX + HEX-G8 carrier | 1824 | 1734 | 1531 | 1482 | 1693 | 1472 | 1439 | 1543 | 1511 |
| A8 Non-covalent DOX + DBCO-G8 carrier | 1490 | 1373 | 1471 | 1580 | 1405 | 1691 | 1420 | 1537 | 1781 |
| K1 Vehicle (ethanol) | 113871 | 128657 | 135872 | 137198 | 129897 | 140012 | 127634 | 121034 | 126764 |
| K2 Untreated control | 113460 | 114348 | 126410 | 135352 | 114453 | 117832 | 135901 | 101387 | 127298 |
| K3 death/max | 112 | 173 | 184 | 234 | 207 | 183 | 115 | 194 | 215 |

*† The per-experiment ATP values for arms A2 and A3 were transposed in the originally recorded table; the row labels, not the measured values, were affected. The direction of the correction is fixed by two read-outs that do not depend on this measurement: the LDH arm of the same panel, run as a separate assay on a separate plate, and the SKBR3 control panel, run on a different cell line (Tables S8–S10). Both rank DOX-ACA-HEX-G8 as the more cytotoxic of the two conjugates, as does the independent 3D dose–response experiment, although the latter is itself an ATP read-out and is therefore separately acquired rather than orthogonal. No assignment other than the corrected one is consistent with all three. The DNA and LDH arms (Tables S6 and S7) and the SKBR3 panel were examined by the same method and required no change.*

*Table S6. AC16 — DNA (biomass, raw) (E = experiment; nine technical values per arm).*

| **Arm** | **E1.1** | **E1.2** | **E1.3** | **E2.1** | **E2.2** | **E2.3** | **E3.1** | **E3.2** | **E3.3** |
| --- | --- | --- | --- | --- | --- | --- | --- | --- | --- |
| A1 DOX·HCl (free doxorubicin) | 82 | 93 | 74 | 113 | 276 | 69 | 102 | 94 | 89 |
| A2 DOX-ACA-HEX-G8 (covalent conjugate) | 375 | 391 | 428 | 382 | 341 | 521 | 296 | 317 | 407 |
| A3 DOX-ACA-DBCO-G8 (covalent conjugate) | 381 | 471 | 362 | 301 | 371 | 324 | 275 | 472 | 361 |
| A4 Carrier HEX-G8 (no DOX) | 432 | 401 | 372 | 327 | 335 | 402 | 377 | 502 | 440 |
| A5 Carrier DBCO-G8 (no DOX) | 473 | 373 | 812 | 381 | 207 | 318 | 309 | 300 | 481 |
| A6 DOX-ACA-N3 (linker–DOX, no peptide) | 104 | 75 | 115 | 167 | 114 | 83 | 96 | 111 | 156 |
| A7 Non-covalent DOX + HEX-G8 carrier | 136 | 72 | 74 | 77 | 129 | 99 | 83 | 94 | 87 |
| A8 Non-covalent DOX + DBCO-G8 carrier | 173 | 111 | 93 | 83 | 87 | 141 | 87 | 127 | 101 |
| K1 Vehicle (ethanol) | 412 | 403 | 371 | 372 | 345 | 291 | 305 | 378 | 438 |
| K2 Untreated control | 472 | 501 | 389 | 367 | 428 | 401 | 372 | 369 | 405 |
| K3 death/max | 15 | 19 | 26 | 34 | 11 | 21 | 27 | 34 | 18 |

*Table S7. AC16 — LDH (raw) (E = experiment; nine technical values per arm).*

| **Arm** | **E1.1** | **E1.2** | **E1.3** | **E2.1** | **E2.2** | **E2.3** | **E3.1** | **E3.2** | **E3.3** |
| --- | --- | --- | --- | --- | --- | --- | --- | --- | --- |
| A1 DOX·HCl (free doxorubicin) | 1735 | 1863 | 1581 | 1683 | 1712 | 1792 | 1634 | 1599 | 1601 |
| A2 DOX-ACA-HEX-G8 (covalent conjugate) | 312 | 591 | 381 | 295 | 481 | 371 | 512 | 475 | 490 |
| A3 DOX-ACA-DBCO-G8 (covalent conjugate) | 272 | 211 | 204 | 178 | 205 | 193 | 252 | 201 | 235 |
| A4 Carrier HEX-G8 (no DOX) | 46 | 51 | 65 | 72 | 39 | 52 | 58 | 64 | 71 |
| A5 Carrier DBCO-G8 (no DOX) | 73 | 78 | 62 | 80 | 64 | 68 | 56 | 72 | 69 |
| A6 DOX-ACA-N3 (linker–DOX, no peptide) | 1471 | 1593 | 1623 | 1728 | 1532 | 1558 | 1572 | 1492 | 1479 |
| A7 Non-covalent DOX + HEX-G8 carrier | 1539 | 1643 | 1387 | 1273 | 1470 | 1523 | 1491 | 1410 | 1582 |
| A8 Non-covalent DOX + DBCO-G8 carrier | 1620 | 1743 | 1572 | 1471 | 1735 | 1701 | 1658 | 1681 | 1535 |
| K1 Vehicle (ethanol) | 67 | 81 | 42 | 39 | 71 | 63 | 71 | 48 | 36 |
| K2 Untreated control | 71 | 64 | 82 | 37 | 39 | 43 | 57 | 63 | 59 |
| K3 death/max | 1845 | 1759 | 1712 | 1693 | 1782 | 1734 | 1634 | 1503 | 1639 |

*Table S8. SKBR3 — ATP (raw) (E = experiment; nine technical values per arm).*

| **Arm** | **E1.1** | **E1.2** | **E1.3** | **E2.1** | **E2.2** | **E2.3** | **E3.1** | **E3.2** | **E3.3** |
| --- | --- | --- | --- | --- | --- | --- | --- | --- | --- |
| A1 DOX·HCl (free doxorubicin) | 1287 | 1175 | 1275 | 1341 | 1280 | 1172 | 1534 | 1389 | 1312 |
| A2 DOX-ACA-HEX-G8 (covalent conjugate) | 22104 | 25340 | 26180 | 23940 | 21865 | 24910 | 23440 | 26124 | 22880 |
| A3 DOX-ACA-DBCO-G8 (covalent conjugate) | 47214 | 49562 | 51340 | 48912 | 46785 | 50112 | 49845 | 51020 | 49099 |
| A4 Carrier HEX-G8 (no DOX) | 112842 | 114562 | 113766 | 114562 | 119792 | 118461 | 112879 | 113561 | 116289 |
| A5 Carrier DBCO-G8 (no DOX) | 112764 | 113562 | 115672 | 111763 | 114783 | 114811 | 112791 | 114582 | 115009 |
| A6 DOX-ACA-N3 (linker–DOX, no peptide) | 1056 | 1028 | 1164 | 1099 | 1174 | 993 | 1002 | 1010 | 1131 |
| A7 Non-covalent DOX + HEX-G8 carrier | 984 | 972 | 1003 | 879 | 1126 | 933 | 1127 | 978 | 991 |
| A8 Non-covalent DOX + DBCO-G8 carrier | 1078 | 845 | 792 | 882 | 912 | 903 | 1029 | 837 | 1114 |
| K1 Vehicle (ethanol) | 111467 | 112765 | 116535 | 115621 | 114370 | 128532 | 117381 | 111279 | 112745 |
| K2 Untreated control | 112341 | 114654 | 115372 | 116243 | 113671 | 114623 | 116498 | 114392 | 114511 |
| K3 death/max | 1001 | 986 | 932 | 867 | 804 | 732 | 936 | 812 | 875 |

*Table S9. SKBR3 — DNA (biomass, raw) (E = experiment; nine technical values per arm).*

| **Arm** | **E1.1** | **E1.2** | **E1.3** | **E2.1** | **E2.2** | **E2.3** | **E3.1** | **E3.2** | **E3.3** |
| --- | --- | --- | --- | --- | --- | --- | --- | --- | --- |
| A1 DOX·HCl (free doxorubicin) | 101 | 97 | 81 | 94 | 87 | 112 | 82 | 91 | 79 |
| A2 DOX-ACA-HEX-G8 (covalent conjugate) | 95 | 82 | 104 | 99 | 83 | 75 | 85 | 89 | 94 |
| A3 DOX-ACA-DBCO-G8 (covalent conjugate) | 105 | 104 | 98 | 75 | 83 | 77 | 93 | 98 | 92 |
| A4 Carrier HEX-G8 (no DOX) | 412 | 501 | 428 | 413 | 387 | 391 | 428 | 401 | 387 |
| A5 Carrier DBCO-G8 (no DOX) | 398 | 375 | 378 | 412 | 403 | 377 | 392 | 415 | 416 |
| A6 DOX-ACA-N3 (linker–DOX, no peptide) | 87 | 92 | 101 | 99 | 92 | 83 | 79 | 94 | 77 |
| A7 Non-covalent DOX + HEX-G8 carrier | 83 | 87 | 99 | 73 | 77 | 87 | 83 | 86 | 102 |
| A8 Non-covalent DOX + DBCO-G8 carrier | 69 | 102 | 98 | 99 | 74 | 89 | 90 | 103 | 87 |
| K1 Vehicle (ethanol) | 445 | 443 | 375 | 488 | 402 | 385 | 345 | 412 | 411 |
| K2 Untreated control | 401 | 472 | 378 | 408 | 432 | 410 | 423 | 325 | 444 |
| K3 death/max | 12 | 74 | 55 | 36 | 81 | 45 | 28 | 49 | 58 |

*Table S10. SKBR3 — LDH (raw) (E = experiment; nine technical values per arm).*

| **Arm** | **E1.1** | **E1.2** | **E1.3** | **E2.1** | **E2.2** | **E2.3** | **E3.1** | **E3.2** | **E3.3** |
| --- | --- | --- | --- | --- | --- | --- | --- | --- | --- |
| A1 DOX·HCl (free doxorubicin) | 1823 | 1777 | 1792 | 1812 | 1685 | 1739 | 1826 | 1739 | 1823 |
| A2 DOX-ACA-HEX-G8 (covalent conjugate) | 345 | 287 | 276 | 212 | 371 | 201 | 356 | 340 | 287 |
| A3 DOX-ACA-DBCO-G8 (covalent conjugate) | 256 | 298 | 305 | 418 | 269 | 276 | 303 | 287 | 312 |
| A4 Carrier HEX-G8 (no DOX) | 41 | 38 | 46 | 51 | 43 | 45 | 53 | 57 | 49 |
| A5 Carrier DBCO-G8 (no DOX) | 55 | 62 | 55 | 68 | 64 | 51 | 56 | 63 | 49 |
| A6 DOX-ACA-N3 (linker–DOX, no peptide) | 1756 | 1741 | 1834 | 1698 | 1837 | 1649 | 1858 | 1749 | 1867 |
| A7 Non-covalent DOX + HEX-G8 carrier | 1801 | 1748 | 1689 | 1638 | 1730 | 1805 | 1836 | 1756 | 1798 |
| A8 Non-covalent DOX + DBCO-G8 carrier | 1745 | 1738 | 1805 | 1800 | 1638 | 1692 | 1748 | 1773 | 1843 |
| K1 Vehicle (ethanol) | 68 | 52 | 42 | 41 | 54 | 61 | 39 | 57 | 59 |
| K2 Untreated control | 43 | 54 | 55 | 48 | 57 | 68 | 52 | 58 | 39 |
| K3 death/max | 1845 | 1820 | 1746 | 1879 | 1723 | 1798 | 1649 | 1738 | 1802 |

**Table S11. Games-Howell post-hoc p values for the control-panel contrasts quoted in the main text (10 µM, 72 h; per-experiment means, n = 3 independent experiments; k = 10 arms per read-out).**

| **Contrast** | **LDH** | **ATP** | **DNA** | **LDH** | **ATP** | **DNA** |
| --- | --- | --- | --- | --- | --- | --- |
|  | **AC16** | **AC16** | **AC16** | **SKBR3** | **SKBR3** | **SKBR3** |
| DOX-ACA-HEX-G8 vs free DOX | 0.002 | 0.001 | 0.012 | <0.001 | <0.001 | >0.99 |
| DOX-ACA-HEX-G8 vs non-covalent mixture (DOX + HEX-G8) | 0.002 | 0.001 | 0.020 | <0.001 | <0.001 | >0.99 |
| DOX-ACA-DBCO-G8 vs free DOX | <0.001 | 0.002 | 0.015 | 0.001 | <0.001 | >0.99 |
| DOX-ACA-DBCO-G8 vs non-covalent mixture (DOX + DBCO-G8) | 0.003 | 0.002 | 0.017 | 0.003 | <0.001 | >0.99 |
| DOX-ACA-HEX-G8 vs DOX-ACA-DBCO-G8 | 0.169 | 0.002 | >0.99 | >0.99 | <0.001 | >0.99 |

*Welch one-way ANOVA across the ten arms: AC16 LDH F(9, 7.5) = 728, p < 0.001; AC16 DNA F(9, 7.3) = 1463, p < 0.001; SKBR3 LDH F(9, 7.7) = 469, p < 0.001; SKBR3 DNA F(9, 7.3) = 5246, p < 0.001. For ATP the Welch F statistic exceeds 10⁶ in both lines because the arms treated with free DOX or the mixtures normalize to ~1% of control with a between-experiment SD below the reporting precision; the test is degenerate in that regime and the F value is not interpretable, so only p < 0.001 is quoted and the effect size (~1% versus 21–82% of control) carries the conclusion. With n = 3 the post-hoc p values below have wide sampling variability; they indicate which contrasts the data support and are not graded evidence. Note that the two conjugates are indistinguishable on AC16 LDH (p = 0.169) and on biomass in both lines (p > 0.99), and that they differ on AC16 ATP (p = 0.002) and SKBR3 ATP (p < 0.001).*

**S4. Cellular uptake in the triple-negative model (MDA-MB-231)**

*Figure S1. Doxorubicin autofluorescence in MDA-MB-231 (triple-negative) cells at an equimolar, sub-lethal 2 µM (24 h). Panels (left to right): vehicle (ethanol) control, free DOX-HCl, DOX-ACA-HEX-G8, and DOX-ACA-DBCO-G8. Free DOX and both conjugates produce clear intracellular red signal, showing that the conjugates are internalized by triple-negative cells even though they are poorly cytotoxic to this model (main text, Table 2). The lack of activity against MDA-MB-231 therefore reflects events downstream of cellular entry rather than a failure of uptake. Red = intrinsic doxorubicin fluorescence (drug accumulation), NOT a viability measure; uniform autocontrast applied.*
